# Restarting iterative projection methods for Hermitian nonlinear eigenvalue problems with minmax property

**DOI:** 10.1007/s00211-016-0804-3

**Published:** 2016-05-14

**Authors:** Marta M. Betcke, Heinrich Voss

**Affiliations:** 1 Department of Computer Science, University College London, Gower Street, London, WC1E 6BT UK; 2 Institute of Mathematics, Hamburg University of Technology, 21071 Hamburg, Germany

**Keywords:** Nonlinear eigenvalue problem, Iterative projection method, Nonlinear Arnoldi method, Jacobi-Davidson method, Minmax characterization, Restart, Purge and lock, 65F15, 15A18, 35P30, 49R50, 65N25

## Abstract

In this work we present a new restart technique for iterative projection methods for nonlinear eigenvalue problems admitting minmax characterization of their eigenvalues. Our technique makes use of the minmax induced *local enumeration* of the eigenvalues in the inner iteration. In contrast to global numbering which requires including all the previously computed eigenvectors in the search subspace, the proposed local numbering only requires a presence of one eigenvector in the search subspace. This effectively eliminates the search subspace growth and therewith the super-linear increase of the computational costs if a large number of eigenvalues or eigenvalues in the interior of the spectrum are to be computed. The new restart technique is integrated into nonlinear iterative projection methods like the Nonlinear Arnoldi and Jacobi-Davidson methods. The efficiency of our new restart framework is demonstrated on a range of nonlinear eigenvalue problems: quadratic, rational and exponential including an industrial real-life conservative gyroscopic eigenvalue problem modeling free vibrations of a rolling tire. We also present an extension of the method to problems without minmax property but with eigenvalues which have a dominant either real or imaginary part and test it on two quadratic eigenvalue problems.

## Introduction

In this work we consider a problem of computing a large number of eigenvalues in an open real interval $J \subset \mathbb {R}$ and the corresponding eigenvectors of the nonlinear eigenvalue problem (NEP)

1 $$\begin{aligned} T(\lambda )x=0, \end{aligned}$$

where $T(\lambda )\in \mathbb C^{n\times n}$ is a family of large and sparse Hermitian matrices for every $\lambda \in J$. We furthermore assume that the eigenvalues of (1) in *J* can be characterized as minmax values of a Rayleigh functional [35]. Such problems routinely arise in simulation of acoustic properties of e.g.vehicles or their parts in order to minimize the noise exposure to the passengers as well as to the environment.

The problem of computing a moderate number of eigenpairs of a nonlinear eigenvalue problem at the beginning of the spectrum has been extensively studied. For minmax admitting problems a Nonlinear Arnoldi method was suggested in e.g. [29] and the Jacobi-Davidson method in [4]. For more general nonlinear eigenproblems iterative projection methods were considered in [1, 7, 11, 14–17, 19–21, 25, 31, 32, 34]. However, the approach in [4, 29] hits its limitations if a large number of eigenvalues (in particular in the interior of the spectrum) of (1) is needed. To algorithmically exploit the minmax property, one has to project the problem under consideration onto a sequence of search spaces, which dimension is growing with the number of the targeted eigenvalue. For a large number of eigenvalues (or eigenvalues in the interior of the spectrum) this naturally requires an excessive amount of storage and computing time.

In this work we propose a new restart technique which allows to project the NEP (1) only onto search spaces of a fixed, relatively small dimension throughout the iteration. The new restart technique can be integrated with iterative projection methods such as the Nonlinear Arnoldi or Jacobi-Davidson method making them capable of computation of a large number of eigenpairs, possibly in the interior of the spectrum. A preliminary version of the local restart technique was published in [18].

The paper is organized as follows. In Sects. 2 and 3 we recapitulate the variational characterization for nonoverdamped nonlinear eigenproblems and the iterative projection methods for their solution. The new restart technique is presented in Sect. 4 along with a strategy for dealing with spurious eigensolutions which are an intrinsic part of the interior eigenvalue computation. The resulting framework for restarting of nonlinear iterative projection methods for interior eigenvalue computation is summarized in Sect. 5. The performance of the restarted methods is demonstrated in Sect. 6 on a range of nonlinear eigenvalue problems with and without minmax property including a real-life industrial gyroscopic eigenvalue problem arising in modeling of the noise radiation from rotating tires. Section 7 concludes the paper with a summary and outlook for future research.

## Variational characterization of eigenvalues

### Definition 1

(*Hermitian Nonlinear Eigenvalue Problem*) Let $T(\lambda )\in {\mathbb C}^{n\times n}$ be a family of Hermitian matrices for every $\lambda$ in an open real interval *J*. As in the linear case, $T(\lambda )=\lambda I-A$, we call the parameter $\lambda \in J$ an *eigenvalue* of $T(\cdot )$, whenever Eq. (1)

$$\begin{aligned} T(\lambda )x=0 \end{aligned}$$

has a nontrivial solution $x\ne 0$, which we call an *eigenvector* corresponding to $\lambda$.

It is well known that all eigenvalues of a linear Hermitian problem $Ax=\lambda x$ are real and if they are ordered, $\lambda _1\le \lambda _2\le \dots \le \lambda _n$, it is possible to characterize them by the minmax principle of Poincaré

### Theorem 1

(Minmax principle of Poincaré) Let $\lambda _1\le \lambda _2\le \dots \le \lambda _n$ be the ordered eigenvalues of $Ax=\lambda x$ then

2 $$\begin{aligned} \lambda _k=\min _{{\mathcal W}\in S_k}\ \max _{w\in {\mathcal W}^1}w^*Aw, \end{aligned}$$

where $S_k$ denotes the set of all *k* dimensional subspaces of ${\mathbb C}^n$ and $\mathcal W^1:=\{w\in \mathcal W\,:\,\Vert w\Vert _2=1\}$ is the unit sphere in $\mathcal W$.

It turns out, that a similar result holds also for a certain type of nonlinear eigenvalue problems.

### Definition 2

(*Rayleigh functional*) Let $f(\lambda ; x):=x^* T(\lambda )x$ be a real function, continuous in J for every fixed $x\ne 0$. Assume that

3 $$\begin{aligned} f(\lambda ;x)=0 \end{aligned}$$

has at most one solution $p(x) \in J$, then (3) implicitly defines a functional *p* on some subset *D* of ${\mathbb C}^n{\setminus }\{0\}$. We refer to *p* as a *Rayleigh functional*, since it generalizes the notation of the Rayleigh quotient in the variational characterization of the eigenvalues of the linear problem.

We furthermore require that

4 $$\begin{aligned} f(\lambda ;x)(\lambda -p(x))>0\quad \text {for every }\lambda \in J\quad \text { and }\quad x\in D\quad \text { with }\lambda \ne p(x), \end{aligned}$$

which is a natural generalization of the requirement that *B* is positive definite for a linear pencil (*A*, *B*).

Under these assumptions a variational characterization in terms of the Rayleigh functional has been considered by various authors. To mention a few, Duffin [9, 10] and Rogers [24] proved the variational principle for the finite dimensional overdamped problems, i.e. problems for which the Rayleigh functional *p* is defined on the entire space ${\mathbb C}^n{\setminus }\{0\}$. Nonoverdamped problems were considered by Werner and the second author [33, 35].

The key to the variational principle is an adequate enumeration of the eigenvalues. In general, the natural enumeration, i.e. the first eigenvalue is the smallest one, followed by the second smallest one etc. is not appropriate (see [33, 35]). Instead, the number of an eigenvalue $\lambda$ of the nonlinear problem (1) is inherited from the number of the eigenvalue 0 of the matrix $T(\lambda )$ based on the following consideration:

### Definition 3

(*Minmax induced numbering of eigenvalues*) Let $\lambda \in J$ be an eigenvalue of the nonlinear problem (1), then $\mu =0$ is an eigenvalue of the linear problem $T(\lambda )x=\mu x$. Therefore there exists $k\in {\mathbb N}$ such that

$$\begin{aligned} 0=\ \max _{{\mathcal W}\in S_k}\ \min _{w\in {\mathcal W}^1}\ w^*T(\lambda )w \end{aligned}$$

or equivalently that 0 is a *k*th largest eigenvalue of the matrix $T(\lambda )$. In this case we call $\lambda$ a *k* *th eigenvalue of* (1).

### Remark 1

For $T(\lambda ):=\lambda B-A$, $B>0$ it follows from the minmax characterization for linear eigenvalue problems that $\lambda$ is a *k*th eigenvalue of $T(\cdot )$ if and only if $\lambda$ is a *k*th smallest eigenvalue of the linear problem $Ax=\lambda Bx$.

### Remark 2

We note that if $T(\lambda )$ is differentiable w.r.t. $\lambda$ and $T'(\lambda )$ is positive definite, then replacing $T(\lambda )x=\mu x$ with the generalized eigenvalue problem $T(\lambda ) x=\kappa T'(\lambda ) x$ yields the same enumeration. This will be used later in Theorem 2.

With this enumeration the following minmax characterization of the eigenvalues of the nonlinear eigenproblem (1) was proved in [33, 35]:

### Theorem 2

(Minmax characterization for eigenvalues of $T(\cdot )$)   For every $x\in D \subset \mathbb {C}^n, x \ne 0$ assume that the real Eq. (3) has at most one solution $p(x)\in J$, and let the definiteness condition (4) be satisfied.

Then the following assertions hold:

(i) For every $k\in {\mathbb N}$ there is at most one *k*th eigenvalue of problem (1) which can be characterized by
  5 $$\begin{aligned} \lambda _k=\min _{\begin{array}{c} {\mathcal W}\in S_k,\\ {\mathcal W}\cap D\ne \emptyset \end{array}} \sup _{w\in {\mathcal W}\cap D}\ p(w). \end{aligned}$$
  Hence, there are at most *n* eigenvalues of (1) in *J*.
(ii) If
  $$\begin{aligned} \lambda _k=\inf _{\begin{array}{c} {\mathcal W}\in S_k,\\ {\mathcal W}\cap D\ne \emptyset \end{array}} \sup _{w\in {\mathcal W}\cap D}\ p(w)\in J \end{aligned}$$
  then $\lambda _k$ is a *k*th eigenvalue of $T(\cdot )$ and (5) holds.
(iii) Assume that for $k<m$ the interval *J* contains the *k*th and the *m*th eigenvalue $\lambda _k$ and $\lambda _m$, then *J* contains all the eigenvalues $\lambda _j \in J, \; j=k,\dots ,m$ and moreover it holds $\lambda _k\le \lambda _{k+1}\le \dots \le \lambda _m$.
(iv) If $\lambda \in J$ and $k\in \mathbb N$ such that problem (1) has a *k*th eigenvalue $\lambda _k\in J$ then it holds that
  $$\begin{aligned} \lambda \left\{ \begin{array}{l}>\\ =\\< \end{array}\right\} \lambda _k \quad \iff \quad \mu _k(\lambda ):=\max _{{\mathcal W}\in S_k}\ \min _{w\in {\mathcal W}^1}\ w^*T(\lambda )w \left\{ \begin{array}{c} >\\ =\\ < \end{array}\right\} 0. \end{aligned}$$

## Iterative projection methods for nonlinear eigenproblems

For sparse linear eigenvalue problems, $Ax=\lambda x$, iterative projection methods are well-established and recognized as a very efficient tool. The key idea is to reduce the dimension of the eigenproblem by projecting it to a subspace of a much smaller dimension. The reduced problem is then handled by a fast technique for dense problems. Of course, this idea can only be successful if the subspace used for projection has good approximating properties w.r.t. some of the wanted eigenpairs, which translates to eigenvalues of the projected matrix being good approximations to the wanted eigenvalues of the large sparse matrix. In iterative projection methods the search subspace is expanded iteratively in a way promoting the approximation of the wanted eigenpairs. The generalizations of iterative projection methods to nonlinear eigenvalue problems were discussed in [1, 4, 7, 11, 15, 17, 19–21, 25, 29, 31, 32, 34]. Two representative examples are the Nonlinear Arnoldi and Jacobi-Davidson methods. Both those methods extend the search subspace targeting a particular eigenvalue. In fact, there are no Krylov subspace methods (i.e. methods which as in linear case would admit a polynomial representation of the search subspace) working directly on the nonlinear eigenvalue problem without linearization. While applying iterative projection methods to general nonlinear eigenvalue problems with the objective to approximate more than one eigenpair, it is crucial to prevent the methods from converging to the same eigenpair repeatedly. In the linear case this is readily done by the Krylov subspace solvers or using partial Schur decomposition [12]. Unfortunately, a similar normal form does not exist for nonlinear eigenvalue problems. While this paper was in review, we became aware of a new approach to avoid repeated eigenpair convergence for general nonsymmetric eigenproblems based on minimal invariant pairs [11]. For nonlinear eigenvalue problems admitting a minmax characterization, in [4, 29] it was proposed to use the induced eigenvalue ordering to remedy the problem. Algorithm 1 outlines a framework for iterative projection methods based on enumeration of the eigenvalues as discussed in Sect. 2.
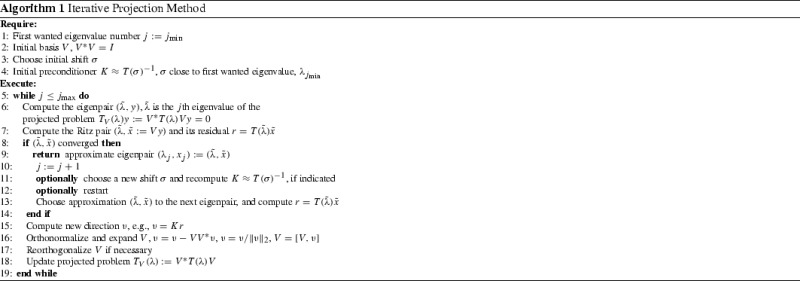


There are many details that have to be considered when implementing an iterative projection method as outlined in Algorithm 1. The comprehensive review is out of scope of this work. Here, we restrict ourselves to only the essentials necessary for motivation and derivation of the local restart technique in Sect. 4. For more detailed discussion we refer the reader to [29, 31, 32].

### Initialization

In order to preserve the numbering of the eigenvalues, the initial basis *V* has to contain at least $j_{\min }$ linearly independent vectors. Let $\mathcal W$ be the invariant subspace of $T(\lambda _{j_{\min }})$ corresponding to its $j_{\min }$ largest eigenvalues then it holds that $z^*T(\lambda _{j_{\min }})z\ge 0$ for every $z\in \mathcal W$, and therefore by Theorem 2 $p(z)\le \lambda _{j_{\min }}$ for every $z\in \mathcal W\cap D$, and $\sup _{z\in \mathcal W\cap D} p(z)=\lambda _{j_{\min }}$. Hence, $\lambda _{j_{\min }}$ is a $j_{\min }$th eigenvalue of the orthogonal projection of $T(\cdot )$ onto $\mathcal W$, and a reasonable choice for the initial space $\mathcal V$ is the corresponding invariant subspace of $T(\tilde{\lambda })$ for some $\tilde{\lambda }$ close to $\lambda _{j_{\min }}$. Likewise, if $T(\cdot )$ is overdamped, then it holds that $z^*T(\lambda _{j_{\min }})z\ge 0$ for every $z\in \text{ span }\{x_1,\dots ,x_{j_{\min }}\}$, where $x_j$ denotes the eigenvector of $T(\cdot )$ corresponding to $\lambda _j$, and the subspace spanned by $x_j$, $j=1,\dots ,j_{\min }-1$ and additionally an approximation to $x_{j_{\min }}$ is also a reasonable choice for the initial search space.

### Solution of a projected nonlinear eigenvalue problem (PNEP)

For nonlinear eigenvalue problem (1) let the columns of $V\in \mathbb C^n$ form a basis of the current search space $\mathcal V \subset \mathbb C^n$. Then it is easily seen that the projected problem

6 $$\begin{aligned} T_{\mathcal V}(\lambda )y:=V^*T(\lambda )Vy=0 \end{aligned}$$

inherits the variational property, i.e. its eigenvalues in *J* are minmax values of the restriction of the Rayleigh functional *p* of $T(\cdot )$ to $D\cap \mathcal V$. Although, in general the enumeration of the eigenvalues of the original problem and the projected problem may differ.

There are many methods for solving small and dense nonlinear eigenvalue problems. For polynomial eigenvalue problems linearization is a natural choice, where the enumeration of eigenvalues in the sense of Sect. 2 can be deduced from the natural ordering of the real eigenvalues of the linearized problem. For general nonlinear eigenvalue problems safeguarded iteration [23] outlined in Algorithm 2 can be used for computing the *k*th eigenvalue of the nonlinear problem. 
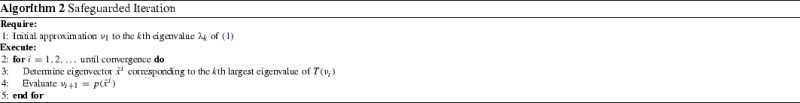


### Subspace expansion

In general two approaches to subspace expansion can be found in the literature: Jacobi-Davidson [4] and Nonlinear Arnoldi [31] type expansion. Both schemes approximate inverse iteration, which is known to provide a direction with high approximating potential to the targeted eigenpair (cubical convergence for symmetric nonlinear eigenproblems if the eigenvalue approximation is updated with the Rayleigh functional).

Let $(\tilde{\lambda }_k,\tilde{x}_k)$ be a currently available approximation to the eigenpair and $r_k = T(\tilde{\lambda }_k)\tilde{x}_k$ its residual. In Jacobi-Davidson the search subspace is expanded by an orthogonal direction $t \perp \tilde{x}_k$ obtained from the following correction equation

7 $$\begin{aligned} \left( I - \frac{T'(\tilde{\lambda }_k)\tilde{x}_k \tilde{x}_k^*}{\tilde{x}_k^*T'(\tilde{\lambda }_k)\tilde{x}_k}\right) T(\tilde{\lambda }_k)\left( I-\frac{\tilde{x}_k\tilde{x}_k^*}{\tilde{x}_k^*\tilde{x}_k}\right) t = -r_k, \quad t \perp \tilde{x}_k. \end{aligned}$$

If (7) is solved exactly we can expect asymptotically cubic convergence. The convergence rates of inexact Newton and Newton-like methods were studied in [27], and it is a common experience that even very coarse solution of (7) is sufficient to maintain a reasonably fast convergence.

The Nonlinear Arnodi method uses the direction of the residual inverse iteration [22]

$$\begin{aligned} v=T(\sigma )^{-1}T(\tilde{\lambda }_k) \tilde{x}_k, \end{aligned}$$

where $\sigma$ is a fixed parameter close to the wanted eigenvalue $\lambda _k$. The Nonlinear Arnoldi method converges linearly, i.e. if $\tilde{x}_k^{i-1}$ and $\tilde{x}_k^i$ are two consecutive iterations with $\Vert \tilde{x}_k^{i-1}\Vert = \Vert \tilde{x}_k^i\Vert =1$ and $\tau = \Vert T(\tilde{\lambda }_k^i)\tilde{x}_k^i\Vert _2 / \Vert T(\tilde{\lambda }_k^{i-1})\tilde{x}_k^{i-1}\Vert _2$ then $\tau =\mathcal O(|\lambda _k-\sigma |)$ (cf. [22]). For Hermitian problems if the eigenvalue approximations are updated with the value of the Rayleigh functional and $\sigma$ is updated with the previous approximation to $\lambda _k$, $\sigma = \tilde{\lambda }_{k}^{i-1}$, [26] the convergence is even quadratic. Moreover, if the linear system $T(\sigma )v=T(\tilde{\lambda }_k) \tilde{x}_k$ is too expensive to solve for *v* we may choose as a new direction $v=KT(\tilde{\lambda }_k) \tilde{x}_k$ with $K\approx T(\sigma )^{-1}$.

### Standard restarting based on global numbering

As the subspace expands in the course of the algorithm, the increasing storage and computational cost of the solution of the projected eigenvalue problem may make it necessary to restart the algorithm and purge some of the basis vectors. To be able to continue determining subsequent eigenpairs the correct enumeration has to be enforced at the restart.

If *J* contains the first eigenvalue $\lambda _1=\min _{x\in D}\ p(x)$, then e.g. the safeguarded iteration for the projected nonlinear problem (6) converges globally, i.e. for any initial vector $x\in {\mathcal V}\cap D$, to the smallest eigenvalue of (6) [23]. Furthermore, if the eigenvectors $x_j$ of the original problem (1) corresponding to the eigenvalues $\lambda _j, \, j=1,\dots ,k$, are contained in $\mathcal V$, then $\lambda _j$ is a *j*th eigenvalue of the projected problem (6), as well. Hence, expanding the search space $\mathcal V$ iteratively, and determining the $(k+1)$st eigenvalue of the projected problems, one gets a sequence of upper bounds to $\lambda _{k+1}$ which (hopefully) converges to $\lambda _{k+1}$. Thus, the eigenvalues of (1) can be determined quite safely one after the other by the iterative projection method starting with an approximation to $x_1$.

If $\inf _{x\in D}\ p(x)\not \in J$ we can modify this approach in the following way. Let *k* be the smallest integer such that $\lambda _k\in J$ where *k* is chosen according to Definition 3. The minimum in (5) is attained by the invariant subspace $\mathcal W$ of $T(\lambda _k)$ spanned by the eigenvectors corresponding to its *k* largest eigenvalues. Hence, if the current search space $\mathcal V$ satisfies ${\mathcal W}\subset {\mathcal V}$ then it is easily seen that $\lambda _k$ is the *k*th eigenvalue of the projected problem (6), i.e. again the numbering of the eigenvalues in the projected and in the original problem coincide, thus the eigenvalues can be determined successively.

In either case, for the numbering to be preserved, the search subspace after restart has to contain the eigenvectors corresponding to all the preceding eigenvalues in *J* and if $\inf _{x\in D}\ p(x)\not \in J$ also appropriate initial vectors, hence the restart requires global information. Notice that we only restart if an eigenvector has just converged since a restart destroys information on the eigenvectors and in particular on the currently iterated one.

### Convergence criterion

In the course of our algorithm we accept an approximate eigenpair $(\tilde{\lambda }_k, \tilde{x}_k)$ as converged, if the residual norm $\Vert T(\tilde{\lambda }_k)\tilde{x}_k\Vert _2 / \Vert \tilde{x}_k\Vert _2$ is small enough. For a linear eigenvalue problem this is just the backward error of the eigenpair. For nonlinear holomorphic eigenvalue problems Szyld and Xue [28] performed a perturbation analysis of simple invariant pairs and derived an error estimate for their approximation. For algebraically simple eigenvalues (i.e. $\det T'(\tilde{\lambda }_k)\ne 0$) their result essentially states that a small residual norm indicates a small backward error, as long as the Jacobian of the augmented system

$$\begin{aligned} \begin{bmatrix}T(\lambda )x\\c^* x-1\end{bmatrix}=0 \end{aligned}$$

is not ill-conditioned at the desired eigenpair $(\lambda _k,x_k)$. Here $c\in \mathbb C^n$ denotes a fixed vector with $c^*x_k\ne 0$.

## A local restart technique

To overcome the problem of the search subspace dimension growing with the number of the sought eigenvalue inherent to global restarts (see Sect. 3.4) we propose to replace the global numbering of the eigenvalues by a local one. As the local numbering is obtained w.r.t. some chosen eigenvalue, only the corresponding eigenvector has to be included into the search subspace after a restart rather than the entire set of preceding eigenvectors or the invariant subspace of $T(\lambda _k)$.

### Local numbering of eigenvalues

Assume that we are given an eigenpair $(\hat{\lambda }, \hat{x})$, $\hat{\lambda }\in J$ and $\hat{x} \in \mathbb {C}^n$, of the nonlinear eigenproblem (1). We refer to such an eigenpair as an anchor. In the following to avoid unnecessary technicalities we assume that $\hat{\lambda }$ is a simple eigenvalue, but all the results can be generalized to allow $\hat{\lambda }$ to be a multiple eigenvalue.

Let $\mathcal V$ be a subspace of $\mathbb C^n$ that contains $\hat{x}$, and let the columns of *V* form a basis of $\mathcal V$. Then, along with the original family of matrices $T(\cdot )$, its projection $T_{\mathcal V}(\cdot ):=V^*T(\cdot )V$ satisfies the conditions of Theorem 2. Therefore the Ritz values of $T(\cdot )$ with respect to $\mathcal V$, i.e. the eigenvalues of the projected eigenproblem (6)

$$\begin{aligned} T_{\mathcal V}(\lambda )y := V^*T(\lambda )V y = 0, \end{aligned}$$

can be enumerated according to Definition 1. In particular, since $\hat{x} \in \mathcal V$, $\hat{\lambda }$ is also an eigenvalue of the projected problem (6), and $\hat{\lambda }$ can be assigned a local number $\ell =\ell (\mathcal V)$ as follows:

The remaining eigenvalues of $T_{\mathcal V}(\cdot )$ (i.e. the Ritz values of $T(\cdot )$ with respect to $\mathcal V$) are given numbers relative to the anchor number, $\ell (\mathcal V)$. We call such a relative numbering local.

#### Example 1

Let $\mathcal V:=\text{ span }\{x_1,x_3,x_7,x_8,x_{10}\}$ where $x_i$ is an eigenvector of (1) corresponding to the *i*th eigenvalue $\lambda _i$. Then the projected problem $T_{\mathcal V}(\lambda )y=0$ has exactly the eigenvalues $\lambda _i$, $i=1,3,7,8,10$ in *J*. For the anchor $\hat{x}:=x_7$ it holds that $\ell =3$, and the local numbers of the subsequent eigenvalues $\lambda _8$ and $\lambda _{10}$ are 4 and 5, respectively.

#### Remark 3

Numerically, the local number of the anchor $\hat{\lambda }$, can be determined as the number of the eigenvalue of the linear problem $T_{\mathcal V}(\hat{\lambda }) y = \mu (\hat{\lambda }) y$ with the smallest absolute value: if $\mu _1\ge \mu _2\ge \cdots$ are its eigenvalues then

$$\begin{aligned} \ell (\mathcal V) := \arg \min _{k = 1,\ldots , \dim \mathcal V} |\mu _k| . \end{aligned}$$

### Spurious eigenvalues

In Example 1, the search subspace $\mathcal V$ has been chosen to contain eigenvectors only, and therefore successive eigenvalues of $T(\cdot )$ with eigenvectors in $\mathcal V$ have consecutive local numbers. However, in a course of iteration, the search subspace will also contain additional vectors besides the eigenvectors which can adversely affect the local numbering. Only for the sake of the following argument let us assume that the nonlinear eigenvalue problem (1) is overdamped such that the Rayleigh functional *p* is defined on $\mathbb C^n{\setminus }\{0\}$. Hence the eigenvectors $X := \{x_1,\dots ,x_n\}$ of $T(\cdot )$ corresponding to the *n* eigenvalues arranged in the ascending order $\lambda _1 \le \lambda _2 \le \dots \le \lambda _n$ form a basis of $\mathbb C^n$.

Let the current search subspace be $\mathcal V_k$, and the anchor pair $(\hat{\lambda }, \hat{x}), \, \hat{x} \in \mathcal V_k$. Assume that from the last restart the method has already computed the eigenvalues $\lambda ^{\mathcal V_k}_{\ell } := \hat{\lambda }< \lambda ^{\mathcal V_k}_{\ell +1} \le \dots \le \lambda ^{\mathcal V_k}_{\ell +j}$ of $T(\cdot )$, which are consecutive eigenvalues of the projected eigenproblem $T_{\mathcal V_k}(\lambda )y=0$ with local numbers $\ell ,\dots ,\ell +j$. After expanding $\mathcal V_k$ to $\mathcal V_{k+1}:=\text {span}\{V_k,v\} =:\text {span}\{V_{k+1}\}$ by some vector *v*, each of $\lambda ^{\mathcal V_k}_{\ell } < \lambda ^{\mathcal V_k}_{\ell +1} \le \dots \le \lambda ^{\mathcal V_k}_{\ell +j}$ remains an eigenvalue of the new projected problem $T_{\mathcal V_{k+1}}(\lambda )y=0$. However, it may happen that $T_{\mathcal V_{k+1}}(\lambda )y=0$ has an additional eigenpair $(\theta , y_{\theta })$ such that $\lambda ^{\mathcal V_k}_{\ell }<\theta \le \lambda ^{\mathcal V_k}_{\ell +j}$.

If $\lambda ^{\mathcal V_k}_{\ell }$ were the smallest eigenvalue of $T(\cdot )$ i.e. $\lambda ^{\mathcal V_k}_\ell = \lambda _1$, then it would be clear that at least one eigenvalue is missing in the interval $(\lambda ^{\mathcal V_k}_\ell ,\lambda ^{\mathcal V_k}_{\ell +j}]$. However, with an anchor in the interior of the spectrum it is possible for the additional Ritz vector, $x_{\theta }:=V_{k+1} y_{\theta }$, that its representation with respect to the eigenbasis *X* of $T(\cdot )$, $x_{\theta }=\sum _i\alpha _i x_i$, contains components $\alpha _i x_i$ such that some of the corresponding eigenvalues $\lambda _i$ are smaller than $\lambda ^{\mathcal V_k}_\ell$ and others are larger than $\lambda ^{\mathcal V_k}_{\ell +j}$ (or larger equal if $\lambda ^{\mathcal V_k}_{\ell +j}$ is a multiple eigenvalue of $T(\cdot )$). We call such a Ritz value $\theta$ a spurious eigenvalue of $T(\cdot )$. The presence of a spurious eigenvalue obviously causes an increase of the local numbers of all the subsequent eigenvalues.

#### Remark 4

(The case $\theta = \hat{\lambda }$) Note that even if $\theta = \hat{\lambda }$ (up to precision to which the eigenvalues are computed), $x_{\theta } \ne \hat{x}$. Hence we can identify such a spurious pair $(\theta , x_{\theta })$ (recall we assumed the anchor $\hat{\lambda }$ to be simple) and enforce the ordering in which $\theta$ precedes $\hat{\lambda }$ so it does not interfere with the local ordering. Therefore, it is sufficient to consider the case $\hat{\lambda }< \theta$.

Our argument took advantage of the existence of an eigenbasis of $\mathbb C^n$, which is a consequence of assuming that the nonlinear eigenvalue problem (1) is overdamped. It is clear, that the same can happen for nonoverdamped problems. The additional complication for nonoverdamped problems is that the linear combination can also contain vectors not in the domain of definition of the Rayleigh functional *p*, which can have the same effect.

Occurrence of spurious eigenvalues is inherent to interior eigenvalue computation. It also happens for linear problems, when no transformation is applied to the eigenproblem to map the eigenvalues from the interior to the lower or upper end of the spectrum. Hence, in order to algorithmically exploit the local numbering we need to find a way to recognize when the local numbering has been obscured by spurious eigenvalues and how to effectively restore it.

### Local restart framework

Algorithm 3 outlines one local restart cycle, which we explain in detail below. 
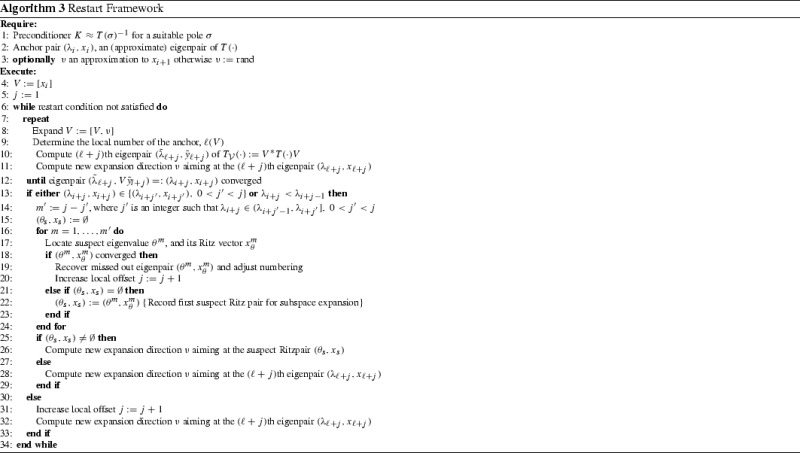


*Lines (1–5): Initialization*   Here, the only difference between the local and and global restarts is the initialization subspace, $\mathcal V_0 := \text{ span } \{\hat{x}, v\}$. For local restarts $\mathcal V_0$ contains only the eigenvector corresponding to the anchor, $\hat{x}$, along with $v \in \mathbb {C}^n$ an approximation to the next eigenvector such that $T_{\mathcal V_0}$ has an eigenvalue $\omega \in J$ with $\omega >\hat{\lambda }$. Starting with $\mathcal V_0$ we determine approximations to the eigenvalue subsequent to the anchor $\hat{\lambda }$ by projecting the nonlinear eigenvalue problem (1) to a sequence of subspaces $\mathcal V_0\subset \mathcal V_1\subset \mathcal V_2\subset \dots$.

*Lines (7–12): Computation of the* $(\ell +j)$ *th eigenpair* $(\lambda _{\ell +j}, x_{\ell +j})$   Let $\mathcal V_k$ be the current search space and $\ell (\mathcal V_k)$ the local number of $\hat{\lambda }$. Note that the number $\ell (\mathcal V_k)$ of the anchor may change in the course of the iteration hence its dependence on $\mathcal V_k$. Suppose that we have successively computed *j* eigenvalues of $T(\cdot )$ in *J*,

$$\begin{aligned} \hat{\lambda }\!=\!\lambda _i=\lambda ^{\mathcal V_k}_\ell<\lambda ^{\mathcal V_k}_{\ell +1} \le \dots \le \lambda ^{\mathcal V_k}_{\ell +j-q-1} < \lambda ^{\mathcal V_k}_{\ell +j-q} = \dots = \lambda ^{\mathcal V_k}_{\ell +j-1} = \lambda _{i+j-1} =:\check{\lambda }, \end{aligned}$$

and let $\check{X}$ be the $1 \le q \le j$ dimensional eigenspace of $T(\cdot )$ corresponding to $\check{\lambda }$. We are now aiming at the next eigenvalue of $T(\cdot )$. To this end, we compute the eigenpair $(\omega ,y_{\omega })$, $\omega \in J$ of the current projected problem $T_{\mathcal V_k}(\cdot )$ with the local number $\ell (\mathcal V_k)+j$. We expand the search space, $\mathcal V_k$ to $\mathcal V_{k+1}$, by a new search direction *v* aiming at the Ritz pair $(\omega ,V_ky_{\omega })$, e.g., $v=KT(\omega )V_ky_{\omega }$ for the Nonlinear Arnoldi method (we hope that, by using such a strategy, as the iteration progresses the search subspace will contain an increasingly significant component of the eigenvector $x_{\ell +j}$). We then solve the new projected eigenproblem $T_{\mathcal V_{k+1} }(\cdot )$ for the Ritz pair with the desired local number $\ell (\mathcal V_{k+1})+j$ and we repeat this iterative process until the Ritz pair with the desired local number has converged (yields a sufficiently small eigenresidual norm, see Sect. 3.5).

#### Remark 5

If $v \in \mathbb {C}^n$ in the initial subspace $\mathcal V_0$ is a poor approximation to $x_{\ell +1}$ and $T_{\mathcal V_0}$ has an eigenvalue $\omega \in J, \, \omega \le \hat{\lambda }$ i.e. $\ell (\mathcal V_0) = \text{ dim }(\mathcal V_0)$ we return the eigenpair with the largest number in the subspace (here the anchor itself, $(\hat{\lambda },\hat{x})$). As $(\hat{\lambda },\hat{x})$ is accurate up to the computational tolerance, its residual $T(\hat{\lambda })\hat{x}$, similarly as a random vector, is expected to be a linear combination of many eigenvectors. The subspace expansion step, e.g. $v=KT(\hat{\lambda })\hat{x}$, then amplifies those eigendirections corresponding to the eigenvalues close to the pole $\sigma$ ($K \approx T^{-1}(\sigma )$) and within a few steps we expect the projected problem $T_{\mathcal V}$ to have an eigenvalue $\omega > \hat{\lambda }$.

*Line 13: Check if a new eigenpair has been computed*   Ideally, the converged Ritz pair $(\omega ,x_{\omega })$ is a new eigenpair of the original problem (1). However, it may happen that the algorithm returns a replicate eigenvalue with number smaller than $i+j$ or a new eigenvalue $\hat{\lambda }< \bar{\lambda }<\check{\lambda }=\lambda _{i+j-1}$ (eigenvalue which has been previously missed out).

From the discussion in Sect. 4.2 we infer that such behaviour occurrs due to the local numbering being altered i.e. one or more additional eigenvalues exist in $(\hat{\lambda },\check{\lambda }]$ correspondingly rising the local number of $\check{\lambda }$. Henceforth we will refer to such eigenvalues as “suspect”. All such suspect eigenvalues can be identified and the missed out eigenvalues can be accepted while the spurious eigenvalues can be treated in the way described below one after the other.

*Lines (14–29): Restoring local numbering*   For the sake of the following argument we assume that the algorithm returned a replicate eigenvalue $\omega = \check{\lambda }$, while any other case follows analogously. Such a repeated convergence of eigenvalues may happen in two cases: (1) $\check{\lambda }$ has a higher geometric multiplicity than *q* (at least $q+1$), and (2) $\check{\lambda }$ is an eigenvalue with multiplicity *q* and $1 \le m' \le q$ additional eigenvalues exist in $(\hat{\lambda },\check{\lambda }]$.

If $\check{\lambda }$ has multiplicity at least $q+1$, which can be ascertained as described in Lemma 1, we simply accept $(\check{\lambda }, x_{\omega } )$ as a newly computed eigenpair and proceed to compute the next eigenvalue whose local number is by 1 larger than the largest local number of $\check{\lambda }$.

#### Lemma 1

If the angle between the eigenspace $\check{X}$, $\text{ dim } \check{X} = q$, and $x_{\omega }$ is different from 0 (in the numerical practice, larger than a prescribed small threshold) or if $\check{\lambda }$ is the $(\ell (\mathcal V^\perp )+j-q)$th eigenvalue of the projected problem

8 $$\begin{aligned} V^{\perp *}T(\lambda )V^{\perp } y=0, \end{aligned}$$

where $\mathcal V$ is the current search space, $V^{\perp }$ denotes a basis of $\mathcal V^\perp$ the orthogonal complement of $\check{X}$ in $\mathcal V$, and $\ell (\mathcal V^\perp )$ the local number of $\hat{\lambda }$, then $\check{\lambda }$ is a multiple eigenvalue (with multiplicity at least $q+1$).

Notice, that the number of columns of $V^\perp$, $\dim (\mathcal V)-q$, is usually quite small and therefore it can be easily verified with safeguarded iteration whether $\check{\lambda }$ is a $(\ell (\mathcal V^\perp )+j-q)$th eigenvalue of the projected eigenproblem (8) or not.

In the second case, there are two possible reasons for the current projected problem having an additional eigenvalue $\theta \in (\hat{\lambda },\check{\lambda }]$ such that the corresponding Ritz pair $(\theta , x_{\theta }) \ne (\lambda _{i+j'}, x_{i+j'}), \, j' = 1,\ldots , j-1$:

1. *Missed out eigenvalue*  An eigenvalue of the original problem (1) in the interval $(\hat{\lambda },\check{\lambda }]$ might have been previously missed out because the corresponding eigenvector $x_{\theta }$ was not sufficiently present in the initial search space $\mathcal V_0$ and might not have been amplified sufficiently in the course of the expansions of $\mathcal V$ until computing $\check{\lambda }$. Afterwards the component of $x_{\theta }$ in the search space $\mathcal V$ has grown large enough to produce the additional eigenvalue $\theta \in (\hat{\lambda },\check{\lambda }]$, and Algorithm 3 yields the eigenvalue $\check{\lambda }$ the $(q+1)$st time with a different local number.
2. *Spurious eigenvalue*  It might be the case that no eigenvalue of (1) is missing in $(\hat{\lambda },\check{\lambda }]$ but the newly produced eigenvalue $\theta$ of the projected problem (6) is a spurious one, i.e. the corresponding Ritz vector $x_{\theta }$ is a linear combination of eigenvectors of (1) corresponding to eigenvalues less than $\hat{\lambda }$ and eigenvalues greater than $\check{\lambda }$ (or greater equal if $\check{\lambda }$ has a higher geometrical multiplicity than computed so far) and possibly some vectors outside of the domain of definition of the Rayleigh functional if the problem is not overdamped.

In both cases we identify the additional eigenvalue $\theta$ and its local number $\ell +j_{\theta }$, and we expand the search space aiming at $(\theta ,x_\theta )$ (in other words, we add a new search direction *v*, which is either $KT(\theta )x_{\theta }$ for the Nonlinear Arnoldi method, or the approximate solution of the Jacobi–Davidson correction Eq. (7) with right–hand side $-T(\theta )x_{\theta }$). Then for the projected problem on such extended subspace $\mathcal V_{\theta }$

9 $$\begin{aligned} T_{\mathcal V_{\theta }}(\lambda ) y =0 \end{aligned}$$

either of the following holds:

- Problem (9) has exactly $j-1$ eigenvalues in $(\hat{\lambda },\check{\lambda }]$, i.e. the additional eigenvalue has left the interval of interest and the numbering in $[\hat{\lambda },\check{\lambda }]$ has been restored.
- There are still *j* eigenvalues in $(\hat{\lambda },\check{\lambda }]$. In this case we repeat the expansion of the subspace until the additional eigenvalue has been moved out from the interval $[\hat{\lambda },\check{\lambda }]$ or the sequence of additional Ritz values has converged to a previously missed out regular eigenvalue, in which case we adjust the enumeration of the eigenvalues and increase *j* by 1.

After the enumeration has been restored we continue with the iterative projection method targeting the eigenvalue with the local number $\ell +j$.

#### Remark 6

In particular if more than one additional eigenvalue exist in $(\hat{\lambda },\check{\lambda }]$, the Algorithm 3 will first identify and recover all missed out eigenvalues. Then the first of the found spurious eigenvalues (i.e. with the smallest local number) will be targeted.

*Lines 31–32: Targeting next eigenvalue*   After convergence of the eigenvalue we may continue the iterative projection method aiming at the $(\ell (\mathcal V_k)+j+1)$st eigenvalue or we may replace the anchor with the newly converged eigenpair and target the eigenvalues subsequent to the new anchor. Since the current search space contains useful information about further eigenvalues it is advisable to continue expanding the search space until the convergence becomes too slow (notice that for the residual inverse iteration the convergence factor $\tau$ depends on the distance between the shift and the wanted eigenvalue) or the dimension exceeds a given bound.

### Automated local restart

For certain problems, the cost to set up a restart, i.e. time for computing the preconditioner, generating the new search space and the projected problem, is relatively high in comparison to the remaining computations. We can further improve the performance allowing the algorithm to balance those time-consuming tasks automatically.

Let $t_{r}$ denote the time for the setup of a restart, and let $t_e^j$ be the time needed for computing the $(\ell +j)$th eigenvalue of problem (1), i.e. *j* denotes the offset of the eigenvalue with respect to the anchor after a restart. Then the total time for computing the first *j* eigenvalues after the restart is $t_t^j = t_r + \sum _{k = 1}^j t_e^k$, and hence the running average time for computing one eigenvalue since last restart is $\bar{t}_e^j = t_t^j / j$. Notice, that as we compute more and more eigenvalues the setup time per eigenvalue decreases.

Let $\alpha \ge 1$ and $N_{\alpha }\in \mathbb N_0$ be parameters depending on the given problem, and we initialize $n_{\alpha }:= N_{\alpha }$. After computing the *j*th eigenpair since a restart we adjust $n_{\alpha }$ in the following way

$$\begin{aligned} n_{\alpha }\leftarrow \left\{ \begin{array}{lll} \min \{N_{\alpha },n_{\alpha }+1\}&{}\quad \text{ if }&{}t_e^j \le \alpha \cdot \bar{t}_e^j\\ n_{\alpha }-1&{}\quad \text{ else }\end{array}\right. \end{aligned}$$

Whenever $n_{\alpha }<0$ we restart the method. The presented strategy compares the time required for convergence of an eigenvalue with the running average time and triggers a restart when the eigenvalue convergence is repeatedly slower by factor $\alpha$ than in average. In particular, if $N_{\alpha }=0$ and $\alpha =1$ we restart the algorithm straightaway when the time for convergence to an eigenvalue exceeds the average time for computing the eigenvalues since the last restart.
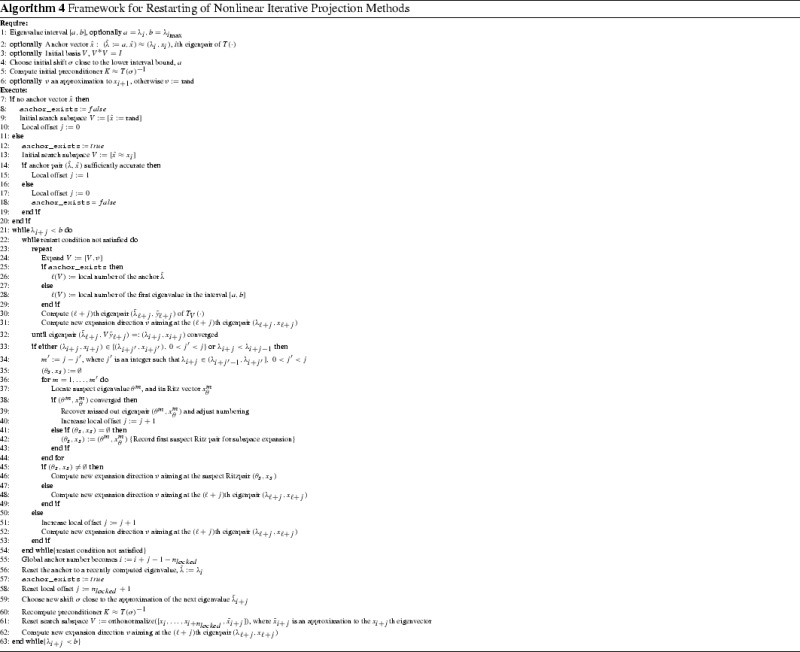


## Framework for restarting nonlinear iterative projection methods

Integrating the local restart with the iterative projection methods, we arrive at the framework for restarting of nonlinear iterative projection methods summarized in Algorithm 4.

An initial anchor pair can be determined for instance with an iterative projection method expanding the search space by $KT(\tilde{\sigma })\tilde{x}_k$ where $\tilde{\sigma }\ne \sigma$ are both fixed shifts close to the wanted eigenvalues, $K\approx T(\sigma )^{-1}$ is a preconditioner, and $\tilde{x}_k$ are the iterates of the projection method aiming at the eigenvalue closest to $\tilde{\sigma }$. Alternatively we could use a direction suggested by the Jacobi Davidson method for the linear eigenproblem $T(\sigma )x = \lambda T'(\tilde{\sigma }) x$ aiming at its smallest eigenvalue in modulus. Obviously, no anchor eigenpair is required if $\inf _{x\in D}p(x)\in J$ and one is looking for eigenvalues at the lower end of the spectrum as the natural enumeration can be used in the first interval. After a restart one of the just computed eigenpairs can serve as an anchor.

More general, for nonlinear eigenvalue problems where the minmax induced ordering and the natural (here ascending) ordering coincide on $[a,b] \subset J$ (e.g. minmax admitting quadratic eigenvalue problem), it is also possible to use one of the bounds of the interval of interest [*a*, *b*] and enumerate the eigenvalues relatively to this bound instead of relatively to an anchor. In such case, we compute the eigenvalues of the projected nonlinear problem $T_{\mathcal V}(\cdot )$ larger or equal *a* until the first restart, when the anchor is reset to an already computed eigenpair. Corollary 1 is a direct consequence of Theorem 2 and it shows how to locate the first eigenvalue of the projected nonlinear problem $T_{\mathcal V}(\cdot )$ larger or equal *a*.

### Corollary 1

Let $(\lambda ^{\mathcal V}_m, y_m)$ be the first eigenvalue of the projected nonlinear problem $T_{\mathcal V}(\cdot )$ in the interval [*a*, *b*]. Then by assumption $\lambda ^{\mathcal V}_{m-1} < a \le \lambda ^{\mathcal V}_m$ and from Theorem 2 it follows

$$\begin{aligned} a> \lambda ^{\mathcal V}_{m-1} \quad\Leftrightarrow & {} \quad \mu _{m-1}(a) = \max _{\mathcal W\in S_{m-1}} \min _{y\in {\mathcal W}^1} y^*T_{\mathcal V}(a) y > 0\\ a \le \lambda ^{\mathcal V}_m \quad \quad\Leftrightarrow & {} \quad \quad \mu _m(a) = \max _{\mathcal W\in S_m} \min _{y\in {\mathcal W}^1} y^*T_{\mathcal V}(a) y \le 0. \end{aligned}$$

Thus, the local number, *m*, of the first eigenpair of $T_{\mathcal V}(\cdot )$ in the interval [*a*, *b*] is the number of the largest nonpositive eigenvalue, $\mu (a) \le 0$, of $T_{\mathcal V}(a)$.

Exactly as before we can target the eigenvalue with the local number *m* and after it converged, the eigenvalue with the local number $m+1$, etc. Theoretically, after the eigenvalue with the *m*th local number converged this could be used as an anchor straight away. However, there is a danger of accepting $\lambda ^{\mathcal V}_m$ as an anchor (hence the first eigenvalue in [*a*, *b*]) prematurely i.e. $\lambda ^{\mathcal V}_m$ is not the first eigenvalue in [*a*, *b*] of the original problem (1) because eigenvalues in $[a, \lambda ^{\mathcal V}_m)$ have been missed out. In this case the algorithm would continue to compute only the eigenvalues larger or equal $\lambda ^{\mathcal V}_m$ permanently missing out the eigenvalues in $[a, \lambda ^{\mathcal V}_m)$. This is less likely to happen if the enumeration w.r.t. the bound *a* is used until the first restart until when the search subspace is large and hence hopefully it includes the first and further consecutive eigenvalues of (1) in [*a*, *b*].

We remark, that the missed out eigenvalues and the spurious eigenvalues have exactly the same effect regardless whether the interval bound *a* or the anchor $\hat{\lambda }$ is used to relatively enumerate the eigenvalues i.e. the local number of the eigenvalues following such missed out/spurious eigenvalue is raised. Hence, they can be dealt with in the same way as described in Sect. 4.3.

Obviously, a very similar strategy can be applied when the anchor pair is not available to the required precision, i.e. its residual norm is above a set tolerance. We incorporated both those important cases in the pseudocode in Algorithm 4.

We might want to keep $n_{locked}$ eigenpairs in addition to the anchor pair at the restart, to minimize the occurrence of spurious eigenvalues. However the benefits have to be traded off against increased cost of the solution of the projected problems due to larger search subspace dimensions.

## Numerical experiments

In this section we demonstrate the performance of the local restarts on a range of nonlinear eigenvalue problems. All the tests were performed with the QARPACK MATLAB package [2] on a desktop machine with two quadcore Intel Xeon X5550, 2.67GHz processors and 48 GB RAM. The LU decompositions and the subsequent system solves for small problems (small gyroscopic problem, “wiresaw1(2000)”, “wiresaw2(2000)”) were performed using MATLAB built-in LU and for large problems (large gyroscopic problem, delay problem, rational problem, “acoustic wave 1d” problem) with the MATLAB’s LU routine with five outputs. For all quadratic problems the projected problems were solved by linearization while for general nonlinear problems with safeguarded iteration.

The results are uniformly presented in terms of elapsed CPU times. We preconditioned the Nonlinear Arnoldi method with the LU factorization of the real part of $T(\sigma )$ where $\sigma$ is a shift not too far away from the wanted eigenvalues. We chose to neglect the imaginary part of $T(\sigma )$ since its influence on the action of the preconditioner is small in our examples, not justifying the extra effort of using complex arithmetic. We updated the LU factorization at each restart.

Table 1 holds the details of the behavior of the Nonlinear Arnoldi method with the local restart strategy described in Sect. 4—Nonlinear Restarted Arnoldi (NRA)—for each of the solved nonlinear eigenvalue problems listed in the first column. The other columns of Table 1 from left to right denote: dim: dimension of the eigenvalue problem; type: type of the eigenvalue problem: gyroscopic, general quadratic, exponential, rational; $\mathcal {R}(\lambda ) \in [a,b]$: interval containing the real part of the wanted eigenvalues; # $\lambda$: number of computed eigenvalues; CPU[s]: CPU time for solution of the nonlinear eigenvalue problem in seconds; PNEP CPU[s]: CPU time for solution of the projected nonlinear eigenvalue problems (PNEPs) in seconds; #iter: number of iterations; #rest: number of restarts. Values for all problems except for the large tire problem are averaged over 10 runs.

**Table 1:** Nonlinear Restarted Arnoldi (NRA) performance for a range of nonlinear eigenvalue problems

NEP	dim	type	$\mathfrak {R}(\lambda ) \in [a, b]$	#$\lambda$	CPU [s]	PNEP CPU [s]	#iter	#rest
wheel	1728	gyro	(11,748,16,820]	100	135.2	71.9	1083.6	26.7
wiresaw1	2000	gyro	[317,629]	100	496.7	111.2	1197.4	21.9
tire	124,992	gyro	[0, 20,000]	388	12.028 [h]	2.87 [h]	5165	22
delay	39,601	exp	[150,250]	75	247.5	58.2	485.1	8.7
fluid-str.	36,040	rat	[10,20]	84	275.6	99.1	464	11.6
wiresaw2	2000	quad	[317,629]	100	850.7	217.1	1060.1	12.4
acoust.1d	30,000	quad	[0, 50]	100	219.5	67.4	618.1	11.1

Values for all problems except for the large tire problem are averaged over 10 runs

### A conservative gyroscopic eigenvalue problem

We consider a conservative gyroscopic eigenvalue problem

10 $$\begin{aligned} T(\lambda )x = \lambda ^2 Mx - i \lambda Gx - Kx = 0 \end{aligned}$$

describing for instance the free vibrations of a rolling tire. It is well known that all its eigenvalues are real and occur in pairs $\pm \lambda$, the corresponding eigenvectors are complex conjugate, and the positive eigenvalues $0 <\lambda _1 \le \dots \le \lambda _n$ satisfy the minmax characterization [10]

$$\begin{aligned} \lambda _i = \min _{{\mathcal W}\in S_i} \max _{w \in {\mathcal W^1}}\ p(w), \end{aligned}$$

where *p*(*x*) is the positive solution of the quadratic equation

$$\begin{aligned} x^* T(\lambda )x = \lambda ^2 x^*Mx - i \lambda x^*Gx - x^*Kx = 0. \end{aligned}$$

#### Qualitative properties of the method

**Fig. 1:**
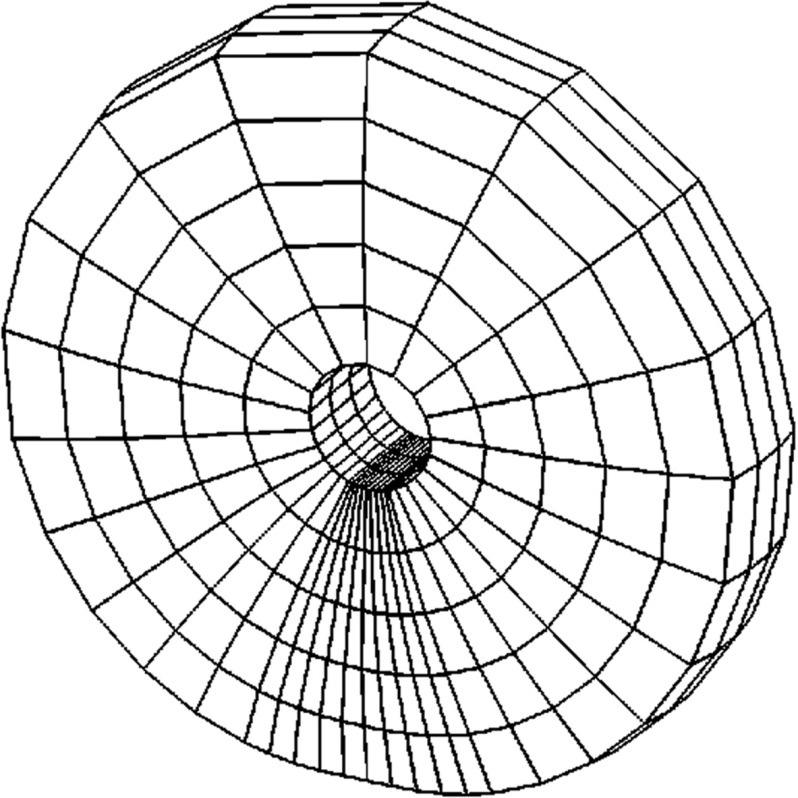
Solid rubber wheel

We start with showing some qualitative behavior of our method on a small example of a wheel, composed of solid elements with Mooney-Rivlin material, see Fig. 1. The wheel is pressed on the track and is rotating at a rate of 50Hz. It is discretized with 450 brick elements with 720 nodes yielding after application of boundary conditions, 1728 degrees of freedom.

For the purpose of comparison we computed all the eigenpairs in the interval [0,16,820] by a globally restarted Nonlinear Arnoldi method. This corresponds to the smallest 200 eigenvalues. In all experiments an eigenvalue was regarded as converged if its relative residual norm was smaller than $10^{-4}$. The preconditioner was recomputed, whenever the ratio $\tau = \Vert T(\tilde{\lambda }_k^s) \tilde{x}_k^s \Vert / \Vert T(\tilde{\lambda }_k^{s-1}) \tilde{x}_k^{s-1}\Vert$ with $\Vert \tilde{x}_k^{s-1}\Vert = \Vert \tilde{x}_k^s\Vert =1$, of two successive residual norms in the last two step $s-1,s$ before convergence of the eigenpair $(\lambda _k, x_k)$ exceeded 0.1. Note, that large $\tau$ indicates that $|\sigma - \lambda _k |$ is to large (see Sect. 3.3). To prevent the search subspace from getting arbitrarily large we used global restarts which were triggered whenever the search subspace dimension exceeded 230, an absolute threshold on the subspace dimension. In the global restart technique we restart the Nonlinear Arnoldi method with an orthogonal basis of the subspace spanned by all eigenvectors computed so far. The total computing time, and the time spent on solving the projected nonlinear problems for the wheel problem are summarized in Table 2.

**Fig. 2:**
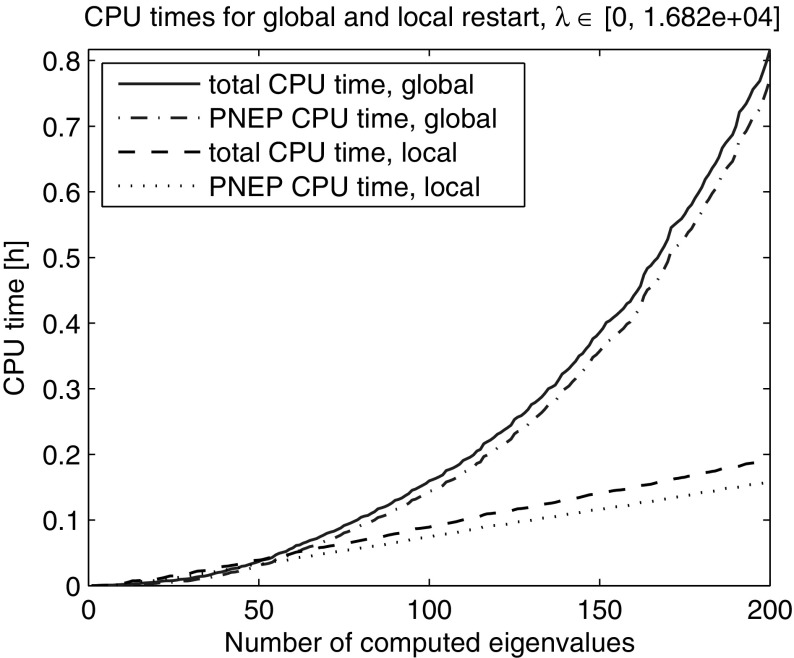
Global versus local restart for first 200 eigenvalues of the gyroscopic wheel problem

In the next experiment we used the same global restart technique, but this time the restart was triggered whenever the dimension of the subspace exceeded the number of the last converged eigenvalue by 30, a relative threshold on the subspace dimension. In this way the average dimension of the search spaces and therefore the time for solving the projected problems were reduced, see Table 2. Plotting the total computing time and the time for solving the projected nonlinear problems in Fig. 2 reveals a super-linear growth. In fact, the CPU time spent on solving the projected eigenproblems itself grows super-linearly, determining the general trend.

**Table 2:** Global restarts with absolute and relative threshold

	CPU [s]	PNEP CPU [s]	#LU	#rest
gl. rest. abs. thres.	11,883	11,721	8	5
gl. rest. rel. thres.	2941	2782	39	41

Comparison of total CPU time, CPU time for solution of the projected nonlinear eigenvalue problems (PNEPs), number of LU decompositions and restarts

**Fig. 3:**
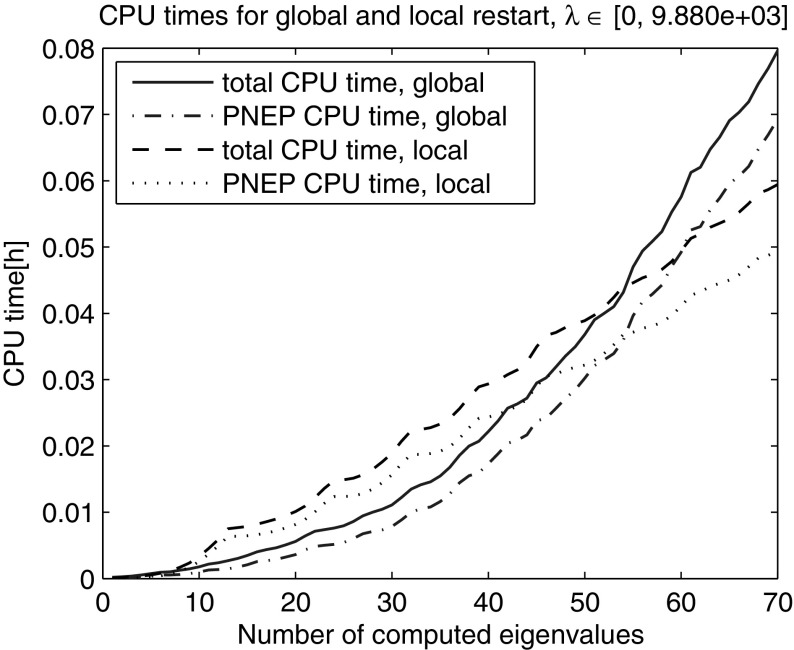
Global versus local restart—zoom into the *lower end* of the spectrum in Fig. 2. When computing the eigenvalues at the beginning of the spectrum in the initial phase the global restart can outperform the local restart with fixed search subspace dimension limit

**Fig. 4:**
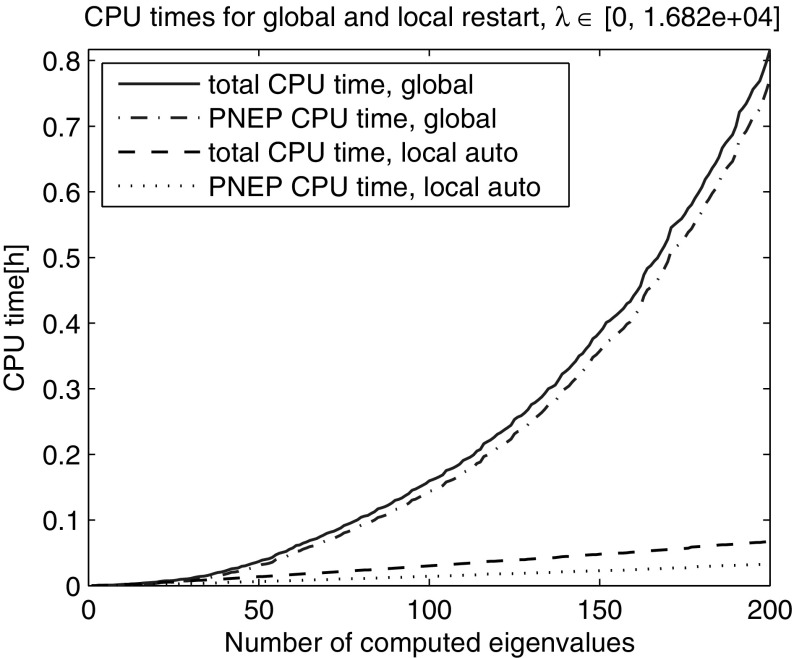
Global versus local restart with automatic balancing for first 200 eigenvalues of the gyroscopic wheel problem

**Fig. 5:**
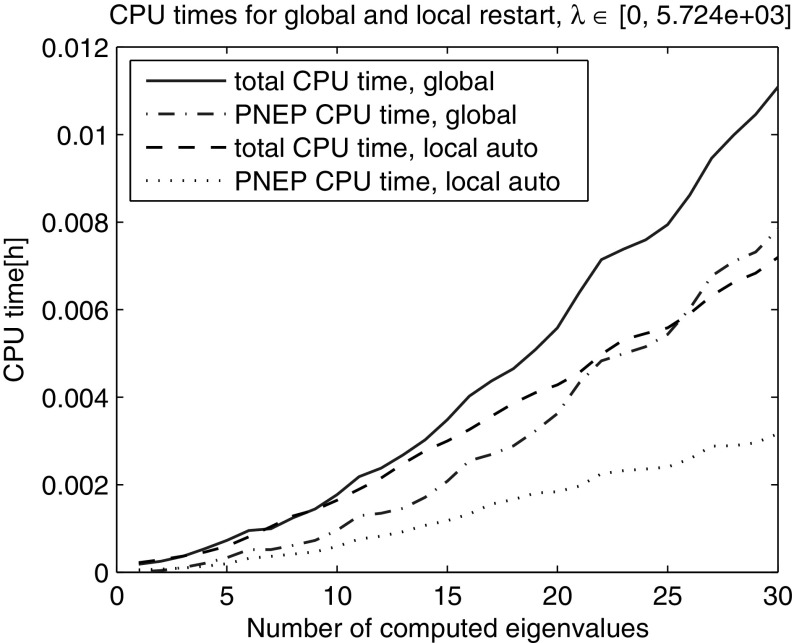
Global versus local restart with automatic balancing—zoom into the *lower end* of the spectrum in Fig. 4. We observe that the balancing effectively restores the superior performance of the local restart over the global restart also in the initial phase

Next, we computed the smallest 200 eigenvalues with Nonlinear Restarted Arnoldi. A restart was triggered whenever the search space dimension exceeded 80 or the convergence rate $\tau$ became larger than 0.5. Only the anchor and the current approximation were kept in the search subspace at restart ($n_{locked}=0$). The experiment was repeated 10 times and the averaged elapsed computing times are shown in Fig. 2. The super-linear time growth has been effectively eliminated through the local restart strategy. The zoom into the lower end of the spectrum reveals that the global restart can outperform the local restart with fixed search space dimension limit in the initial phase when all the eigenvalues from the beginning of the spectrum are computed (Fig. 3). However, as it can be seen in Figs. 4 and 5 the local restart with automatic balancing outperforms the global restart also in the initial phase.

**Fig. 6:**
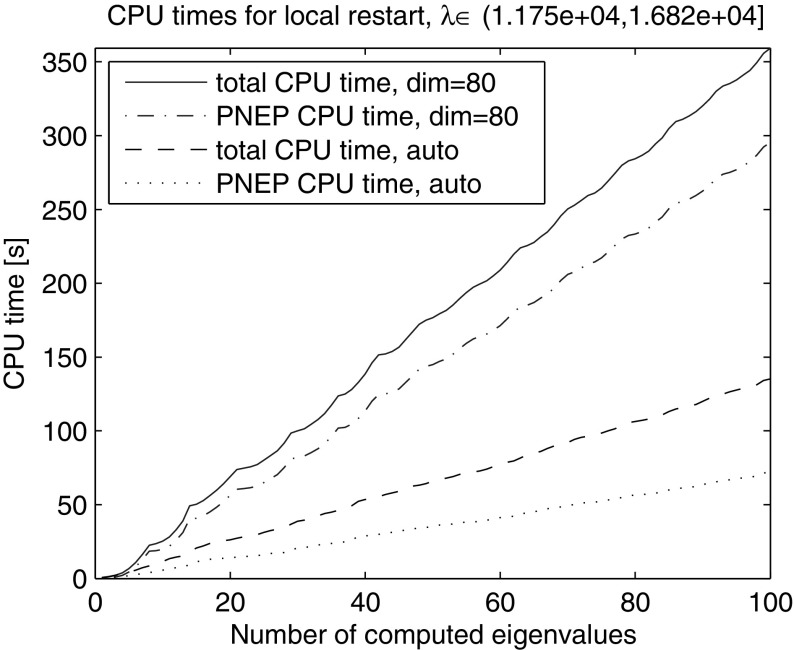
Local restart with automatic balancing for eigenvalues in $(\lambda _{100} = 11{,}748, 16{,}820]$ of the gyroscopic wheel problem (eigenvalues $\lambda _{101},\ldots , \lambda _{200}$)

**Fig. 7:**
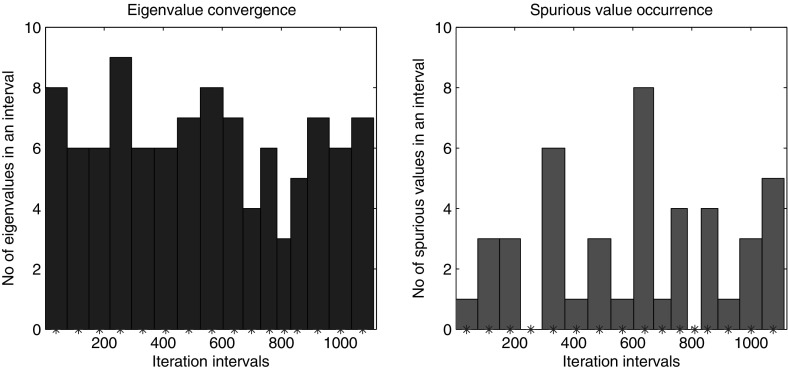
Histogram of *left* eigenvalue convergence, *right* spurious value occurrence per interval between consecutive restarts in one run of computation of eigenvalues in (11,748, 16,820] of the gyroscopic wheel problem

**Fig. 8:**
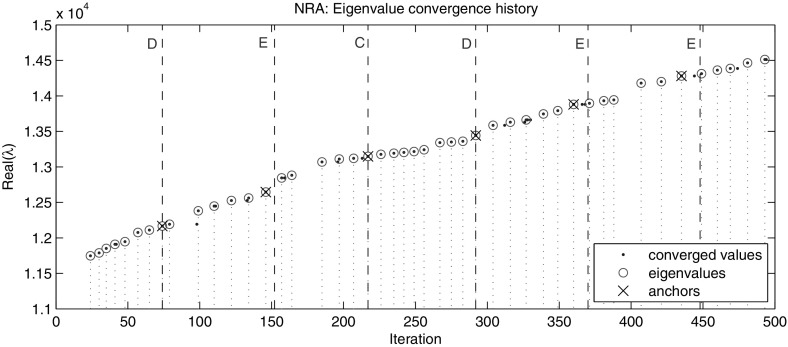
Eigenvalue convergence history throughout first 500 iterations of NRA while computing eigenvalues in (11, 748, 16, 820] of the gyroscopic wheel problem

The outstanding advantage of the local restart strategy is its ability of computing eigenvalues in an interval in the interior of the spectrum without the need of computing all the preceding eigenpairs. Using the same local restart strategy we computed all the eigenpairs in $(\lambda _{100}=11{,}748, 16{,}820]$ which corresponds to $\lambda _{101},\dots ,\lambda _{200}$ using the eigenpair $(\lambda _{100}, x_{100})$ as the initial anchor. Again for reproducibility of the results we repeated the experiment 10 times. As expected the averaged computing time has been approximately halved from 700 s to 350 s, Fig. 6. To illustrate typical behavior of the method in more detail, in Fig.  7 for just one run of the experiment we plotted histograms of the computed eigenvalues and of the occurrences of the spurious eigenvalues in each of the intervals between the consecutive restarts. The corresponding eigenvalue convergence history throughout first 500 iterations is depicted in Fig. 8. The dots not encircled pin down the occurrence of the spurious values during the iteration e.g. in iterations 98, 159, 213 or 312 in Fig. 8 (cf. histogram in Fig. 7).

**Fig. 9:**
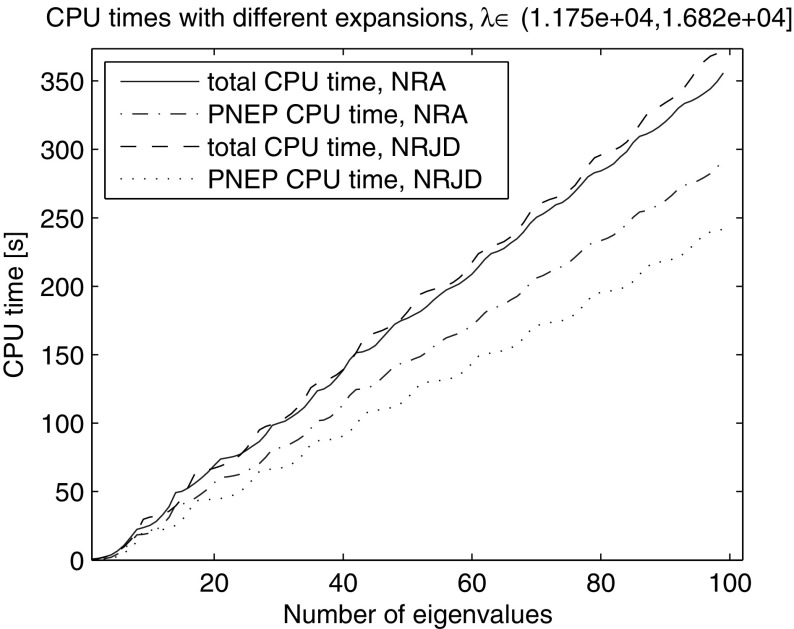
CPU time of NRA and NRJD for computation of eigenvalues in $(\lambda _{100} = 11{,}748, 16{,}820]$ of the gyroscopic wheel problem without automatic balancing

The automated restart strategy described in Sect. 4.4, can be used to let the algorithm balance the limit on the search subspace size on fly. Using the automated restart with $\alpha =1$ and $N_{\alpha }=1$ further reduced the average CPU time to about 130 s, see Fig. 6.

Figures 2, 3, 4, 5 and 6 demonstrate that using the local restart technique, the cost for computing one eigenvalue is approximately the same throughout the iteration, no matter what is the eigenvalue number. Thus we conclude that the new local restart technique effectively eliminates the super-linear CPU time growth with the number of computed eigenvalues and hence constitutes an efficient method for computing eigenvalues in the interior of the spectrum.

For the purpose of performance comparison of Nonlinear Arnoldi (NA) and Jacobi-Davidson (JD) type subspace expansions, we computed all the eigenpairs of the wheel problem in the interval $(\lambda _{100}=11{,}748, 16{,}820]$ using both subspace expansions. Each of the experiments was repeated 10 times and the performance figures were averaged through 10 runs. The automatic balancing was switched off, to focus on the effect of subspace expansion only. In both cases the methods consistently found all eigenvalues. Nonlinear Restarted Arnoldi (NRA) needed on average 1082.6 iterations and 14 restarts (15 LU factorizations), while Nonlinear Restarted Jacobi-Davidson (NRJD) 876.9 and 11 (12), respectively. Nonetheless, the NRA variant is still slightly faster in terms of the total CPU time, see Fig. 9. This is due to an JD expansion step being more expensive than an NA expansion step.

The here used preconditioner (LU decomposition of $K-\sigma ^2M$) remains of reasonable quality in the spectrum of interest. The results are in line with our general experience that Nonlinear Arnoldi method is faster whenever a good quality preconditioner is available, while Jacobi-Davidson method is more robust with respect to poor preconditioning [34].

#### NLEVP “wiresaw1” gyroscopic QEP

As a second example we solve the gyroscopic problem arising from the vibration analysis of a wiresaw, “wiresaw1” from the NLEVP collection [6] of dimension 2000 and with the NLEVP default value of the wire speed parameter $v = 0.01$. The gyroscopic matrix *G* for this problem is not sparse, hence the moderate choice of problem dimension. In formulation (10) all the eigenvalues are real, and are growing by approximately $\pi$ increment from one eigenvalue to the next.

**Fig. 10:**
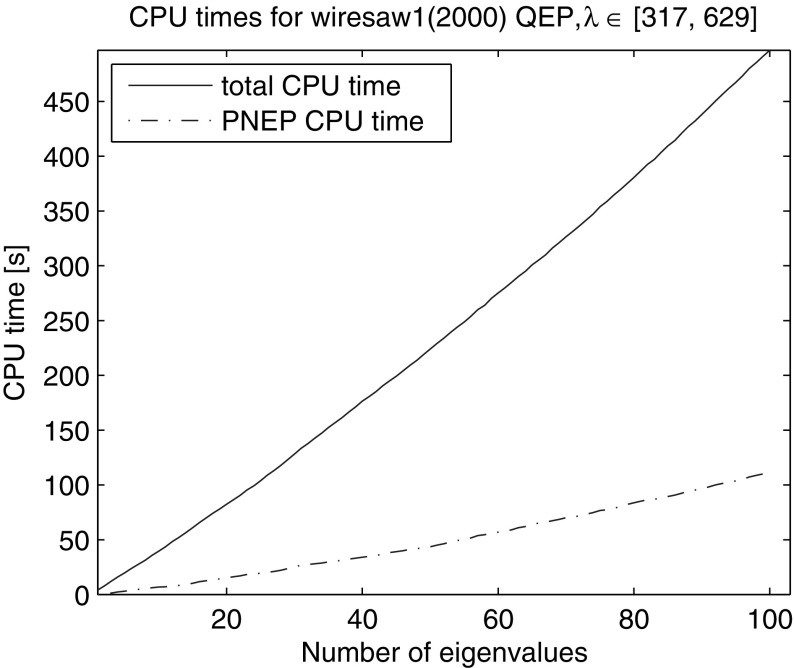
CPU time of NRA for computation of eigenvalues in [317, 629] of the NLEVP “wiresaw1” problem of dimension 2000

We computed all 100 eigenvalues in the interval [317, 629]. The algorithm was initialized using the lower bound of the interval rather than an anchor. The relative residual tolerance was chosen to $10^{-4}$, the maximal subspace dimension to 120, the number of locked eigenvectors after the restart $n_{locked} = 0$ and the slowest admissible convergence rate $\tau = 0.5$. We used automated restarts with $\alpha =1$ and $N_{\alpha } = 1$. Figure 10 shows the CPU time and the time for solution of the projected nonlinear problems averaged over 10 runs. The method took on average 1197 iterations with 22 restarts (23 LU decompositions) to compute the 100 eigenvalues. The average total CPU time was 497 s and the time for solution of the nonlinear projected problems 111 s (q.v. Table 1).

#### Large sparse gyroscopic QEP

**Fig. 11:**
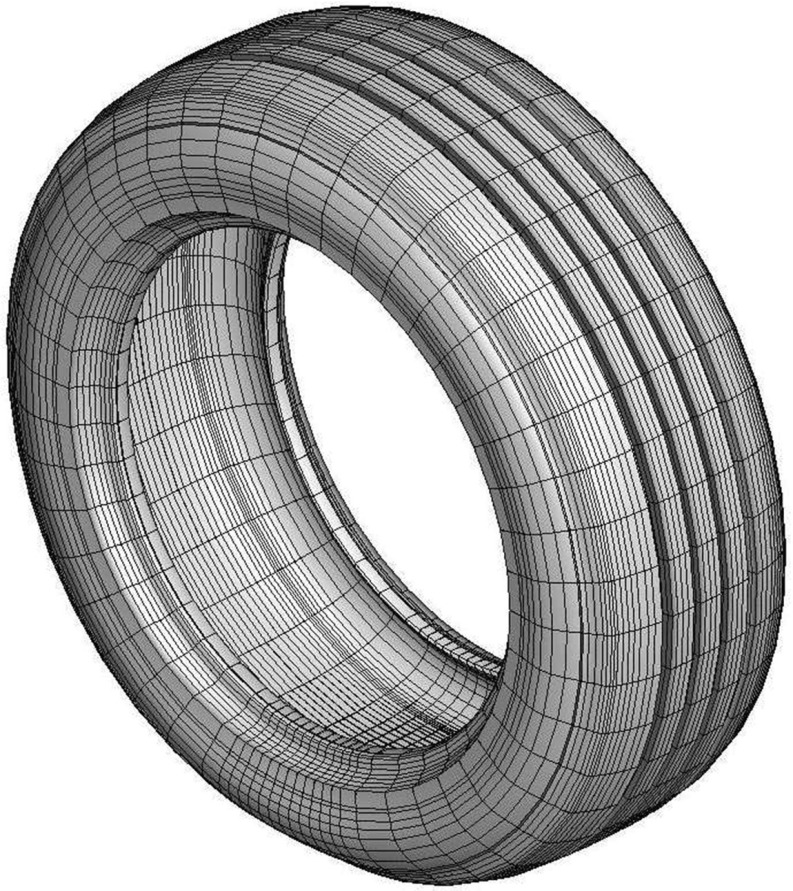
Continental AG 205/55R16-91H tire

Our third example is a tire 205/55R16-91H cf. [19] (see Fig. 11) provided by Continental AG. The tire is discretized with 39,204 brick elements and 42,876 nodes. The nodes at the wheel rim are fixed resulting in 124,992 degrees of freedom. To account for a complex structure of the tire, 19 different materials are used in the finite element model. The model includes the stress approximating the air pressure in the tire. The tire is pressed on the track and is rotating at a rate corresponding to a vehicle speed of 50 km/h.

We used Nonlinear Restarted Arnoldi method with MATLAB’s five output LU routine as a preconditioner and set the tolerance for the relative residual norm to be $10^{-6}$ and the maximal search subspace dimension to 300. After each restart, only the anchor vector and the next approximation were kept in the subspace (i.e. $n_{locked}=0$). The preconditioner was recomputed after at most 300 iterations, subject to residual norm ratio of at most $\tau = 0.9$ and automatic balancing with $N_{\alpha }=1$ and $\alpha =1$.

**Fig. 12:**
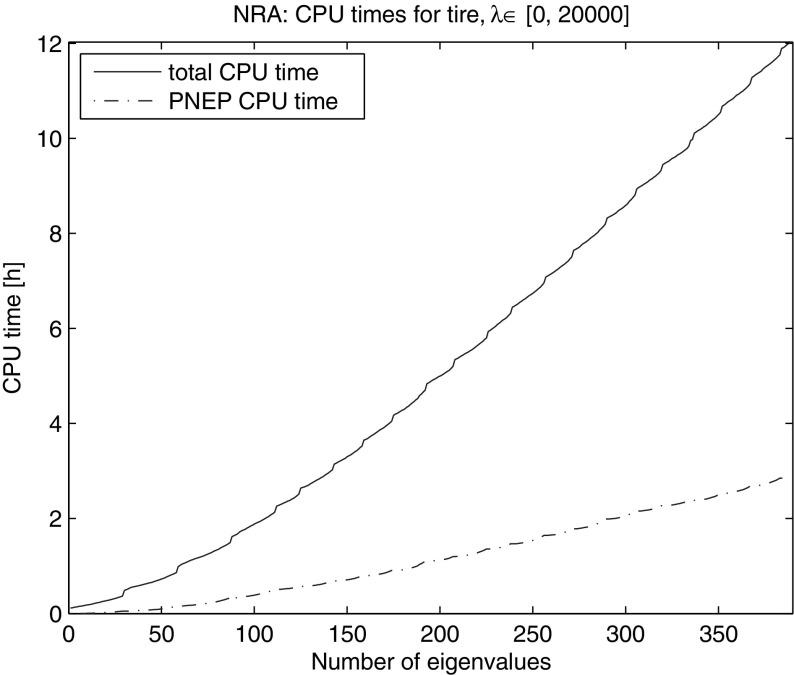
CPU time for NRA for eigenvalues in [0, 20,000] of the gyroscopic tire 205/55R16-91H problem

We computed all the eigenvalues in the interval [0, 20, 000]. NRA needed 5165 iterations and 22 restarts (23 LU factorizations) to find all 388 eigenvalues in this interval. Figure 12 shows the total CPU time and the CPU time for solving projected nonlinear eigenvalue problems. We observe a slight increase in CPU time per eigenvalue, while we compute the eigenvalues at the lower end of the spectrum, which saturates at about 150th eigenvalue. Here, the reason is an increasing occurrence of spurious eigenvalues in proportion to the number of computed eigenvalues in the initial phase. For the eigenvalues higher in spectrum this effect settles, resulting in approximately constant time per eigenvalue computation. All but one restart were triggered through our automatic balancing strategy, demonstrating its effectiveness.

In this example we observed an increased occurrence of spurious values after the restarts. This leads us to believe that retaining some of the previously computed subspace after the restart may help to alleviate this effect, like for instance keeping further $n_{locked}$ eigenvectors along with the anchor in the local basis. Any benefits however, have to be traded off against increased computational cost due to larger search space dimensions.

We believe that the key to the optimal performance is to balance the subspace growth with the occurrence of spurious eigenvalues. An optimal strategy may include adapting the number of eigenvectors kept in the local basis along with the anchor, $n_{locked}$, in dependence of e.g. frequency of occurrence of spurious eigenvalues in the previous interval.

### General nonlinear eigenvalue problems

The following two problems are non-quadratic nonlinear eigenvalue problems. Thus the projected problems are solved by the safeguarded iteration (Algorithm 2). For a general nonlinear function, we do not have an explicit formula for its zeros (and hence for the Rayleigh functional) as it was the case for the quadratic eigenvalue problem but we have to revert to Newton iteration.

In both cases the method was initialized using the lower bound of the interval. The relative residual tolerance was $10^{-6}$, maximal subspace dimension 80, the slowest admissible convergence rate $\tau = 0.5$ and the automatic restart parameters $\alpha =1$ and $N_{\alpha }=1$. We report average performance values over 10 runs.

#### Delay exponential NEP

We consider the following delay differential equation on a square domain [13]

$$\begin{aligned} u_t(\xi ,t)=\Delta u(\xi ,t)+a(\xi )u(\xi ,t)-b(\xi )u(\xi ,t-2),\;\; \xi \in \Omega :=[0,\pi ]^2,\;\; t\ge 0 \end{aligned}$$

with Dirichlet boundary conditions $u(\xi ,t)=0, \; \xi \in \partial \Omega , \; t\ge 0$ and $a(\xi )=8\sin (\xi _1)\sin (\xi _2)$ and $b(\xi )=100|\sin (\xi _1+\xi _2)|$. Using the ansatz $u(\xi ,t)=e^{\lambda t}v(\xi )$ and discretizing the Laplace operator on a uniform grid with step size $\pi /200$ by the 5-point stencil finite difference approximation, we obtain the nonlinear eigenvalue problem

$$\begin{aligned} T(\lambda )x=\lambda x+Ax+e^{-2\lambda }B x=0 \end{aligned}$$

of dimension 39601. *B* is a diagonal matrix corresponding to values of the function $b(\xi _1,\xi _2)$ and *A* is the negative sum of a diagonal matrix with entries corresponding to values of the function $a(\xi _1,\xi _2)$ and the 2-D discrete Laplacian.

**Fig. 13:**
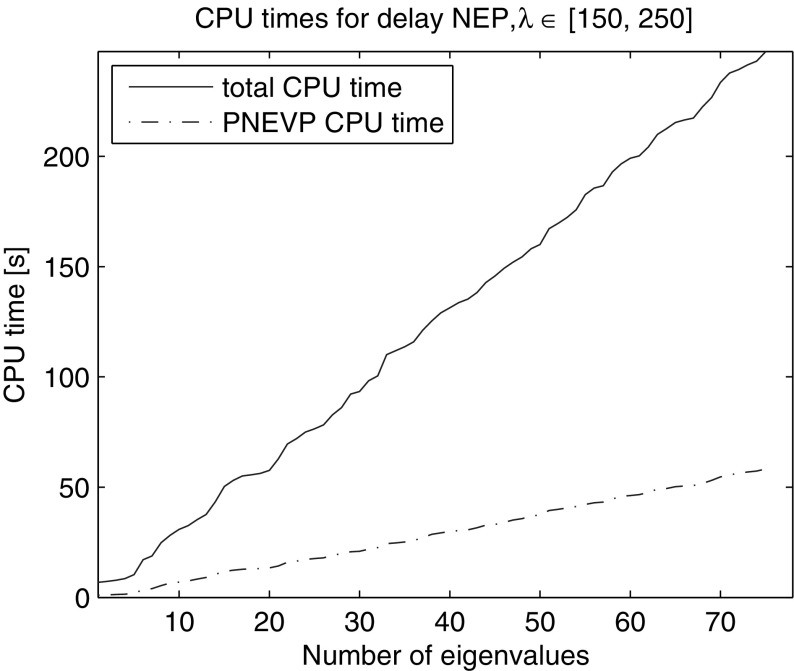
CPU time of NRA for computation of eigenvalues in [150, 250] of the exponential delay problem of dimension 39,601

Due to the symmetry of the problem in $\xi _1$, $\xi _2$ (Laplacian is symmetric on a square domain $\Omega$ and $a(\xi _1,\xi _2) = a(\xi _2,\xi _1), b(\xi _1,\xi _2) = b(\xi _2,\xi _1)$) the problem has double eigenvalues. To avoid missing out eigenpairs, at each restart we locked the preceding eigenvector along with the anchor in the search subspace, $n_{locked} = 1$. We computed all 75 eigenvalues in the interval [150, 250]. Figure 13 shows the linear dependence of the CPU times on the number of computed eigenvalues. On average NRA method took 485.1 iterations with 8.7 restarts (9.7 LU decompositions) over 247.5 s, 58.2 s of which were spent on solution of the projected problems, Table 1.

For problems with double or higher multiplicity eigenvalues, it may be beneficial to consider extension of the restart strategy to block versions of the nonlinear Arnoldi and Jacobi-Davidson methods. Block methods by design are well suited for problems with multiple or clustered eigenvalues, as an entire subspace is iterated simultaneously. In principle, the local restarts can be used within block methods, when we simply iterate a number of eigenpairs, *q*, with consecutive local numbers, say $k, k+1,\dots , k+q-1$ instead of one. At each iteration, all the spurious eigenvalues which disturb the local ordering in the entire interval $(\lambda _{\ell }, \lambda _{\ell + k+q-1}]$ would have to be removed which again could be done using block operations. A large number of numerical tests would be necessary to access whether such restarted block method has a significant advantage over the single vector version. A serious discussion of such extension is beyond the scope of the current paper.

#### Fluid structure interaction rational NEP

Our second problem is a rational eigenvalue problem

11 $$\begin{aligned} Kx=\lambda Mx+\sum _{j=1}^k\frac{\lambda }{\sigma _j-\lambda }C_j x, \end{aligned}$$

where $K,M\in \mathbb R^{n\times n}$ are symmetric and positive definite, $C_j\in \mathbb R^{n\times n}$ are matrices of small rank $r_j$, and $0<\sigma _1<\sigma _2<\dots <\sigma _k$ are given poles. Problems of this type arise for example in free vibrations of tube bundles immersed in a slightly compressible fluid [8].

In each of the intervals $J_j:=(\sigma _{j-1},\sigma _j)$, $j=1,\dots ,k+1$ with $\sigma _0=0$, $\sigma _{k+1}=\infty$, problem (11) satisfies the conditions of the minmax characterization and in each interval the eigenvalues have consecutive numbers [30].

The considered matrix problem is a finite element discretization of an elliptic cavity with 9 emerged tubes with 36040 degrees of freedom, it has 9 poles, $\sigma _j = j, \, j = 1,\dots , 9$ and $\text{ rank } C_j = 2, \, j=1,\dots ,9$ [3].

**Fig. 14:**
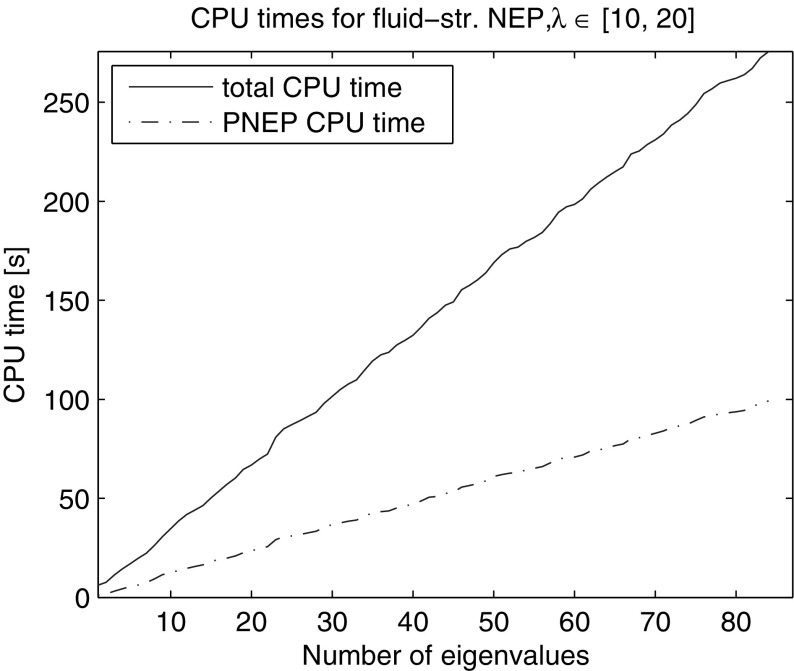
CPU time of NRA for computation of eigenvalues in [10, 20] of the rational fluid structure interaction problem of dimension 36,040

Using the search subspace with only the anchor locked i.e. $n_{locked} = 0$, we computed all 84 eigenvalues in the interval [10, 20]. Figure 14 shows the average total CPU time and CPU time for solution of the projected nonlinear problems with safeguarded iteration. The algorithm took on average 464 iterations with 11.6 restarts (12.6 LU decompositions) over average total CPU time of 275.6 s, 99.1 s of which were spent on solution of projected nonlinear problems.

**Fig. 15:**
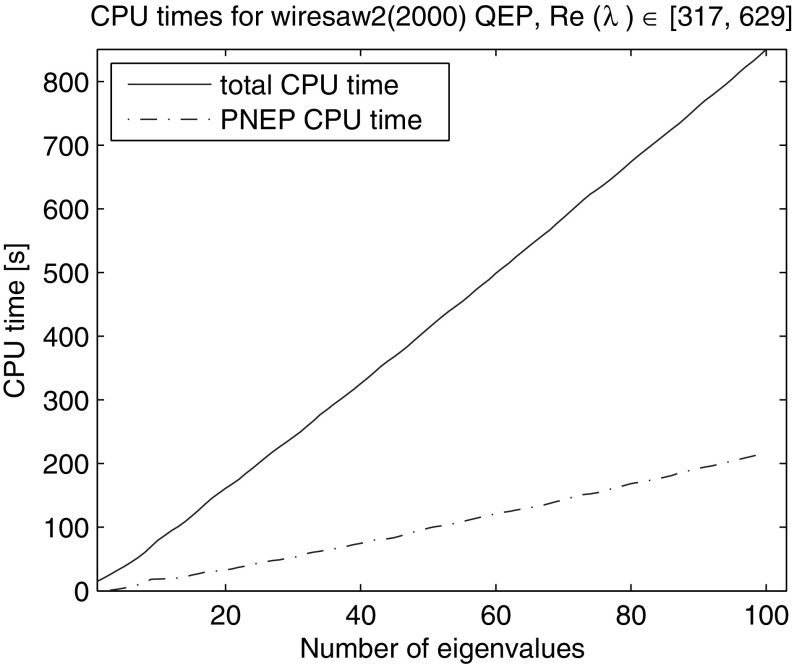
CPU time of NRA for computation of eigenvalues with real part in [317, 629] of the NLEVP “wiresaw2” problem of dimension 2000

### Quadratic eigenvalue problems with eigenvalues with dominant real part

The described local restart procedure hinges upon the minmax property of the nonlinear eigenvalue problem. However, observe that if $(\lambda ,x)$ is an eigenpair of the nonlinear problem $T(\lambda ) x =0$ with a complex eigenvalue, $\lambda \in \mathbb C$, it holds that $\mu =0$ is an eigenvalue of the linear problem $T(\lambda ) x = \mu x$ and also of $T_{\mathcal V}(\lambda ) y = \mu y$ if $x \in \mathcal V$. As there is no natural order in the complex plain, in general we cannot infer the number of the eigenvalue. However, if the eigenvalues have a dominant real part $\mathfrak {R}(\lambda ) \gg \mathfrak {I}(\lambda )$ (or equivalently up to a transformation dominant imaginary part), they can be ordered with respect to the dominant part. Furthermore, this ordering is inherited by the projected problem. If we can solve the projected nonlinear problem for the complex eigenpair $(\lambda , y)$ (e.g. well known issues with convergence of Newton method for complex zeros) we can proceed as in the real case but where the eigenvalues are enumerated w.r.t. the ascending real part. In particular in the polynomial case, the projected polynomial problems can be effectively solved by linearization.

We used such ordering to solve two quadratic eigenvalue problems from the NLEVP collection [5] which eigenvalues have a dominant either real or imaginary part. The projected problems were solved using linerization and the method was initialized using the lower bound of the interval containing the dominant part of the wanted eigenvalues.

#### NLEVP “wiresaw2” QEP

We consider a quadratic eigenvalue problem arising from the vibration analysis of a wiresaw including the effect of viscous damping, “wiresaw2” problem from NLEVP collection [6]

$$\begin{aligned} T(\lambda )x = \lambda ^2 Mx - i\lambda Cx - Kx. \end{aligned}$$

We chose the dimension of 2000 and NLEVP default values of the wire speed and damping parameters, $v = 0.01$ and $\eta = 0.8$, respectively. For this problem both the matrices *C* and *K* are not sparse, hence the relatively small problem dimension. The real parts of the eigenvalues are approximately equal to the corresponding eigenvalues of the “wiresaw1” problem with the same dimension and value of the parameter *v*, and the imaginary part of all eigenvalues is a constant equal to 0.8.

As for “wiresaw1” problem we computed all 100 eigenvalues with the real part in the interval [317, 629] using the same initialization and solver parameters. Figure 15 shows the total CPU time and the CPU time for solution of the projected quadratic problems. While the solution for each complex eigenvalue takes longer than for the corresponding eigenvalue of the real problem, the qualitative property that the method needs approximately equal CPU time per eigenvalue regardless of its location in the spectrum is preserved. On average the solver took 1060 iterations in 851 s, 217 s of which were spent solving projected quadratic problems with 11.1 restarts corresponding to 12.1 LU factorizations.

#### NLEVP “acoustic wave 1D” QEP

We consider a quadratic eigenvalue problem

12 $$\begin{aligned} T(\lambda )x = \lambda ^2 Mx + \lambda Cx + Kx \end{aligned}$$

arising from a finite element discretization of a 1D acoustic wave equation with mixed Dirichlet and impedance boundary conditions, “acoustic wave 1d” problem from NLEVP [6]. The matrices *K*, *M* are real symmetric and *C* is a low rank complex diagonal matrix dependent on the impedance parameter. For the formulation (12) all the eigenvalues lie in the upper half of the complex plane and have a dominant real part.

**Fig. 16:**
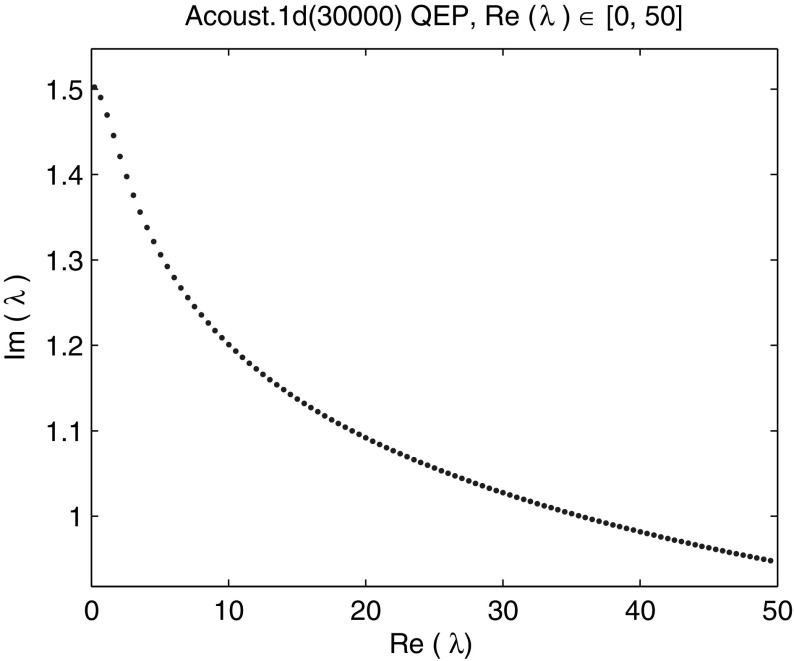
Eigenvalues with the real part in [0, 50] of the NLEVP “acoustic wave 1d” quadratic eigenvalue problem of dimension 30,000

**Fig. 17:**
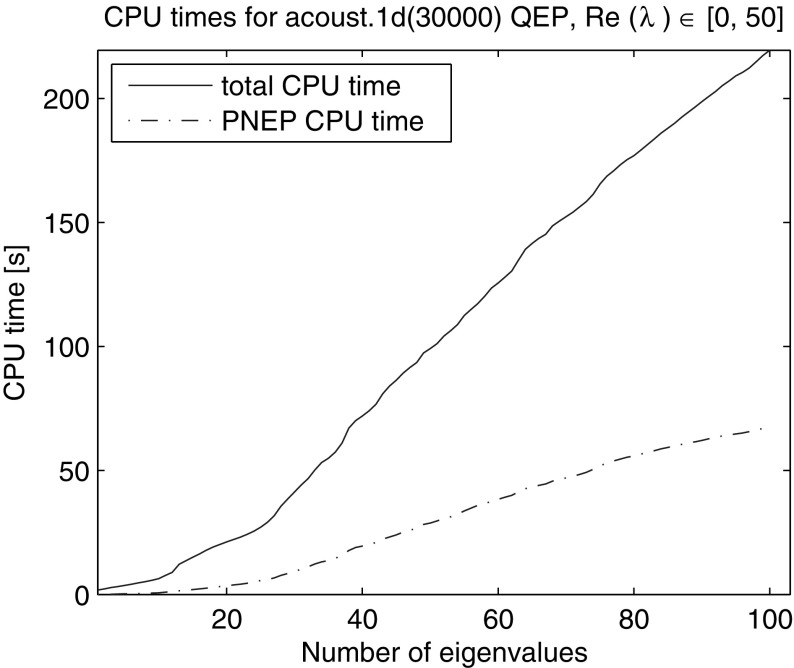
CPU time of NRA for computation of eigenvalues with real part in [0, 50] of the NLEVP “acoustic wave 1d” quadratic eigenvalue problem of dimension 30,000

Using NLEVP default value of the impedance parameter $\zeta =1$ we generated a matrix problem of dimension 30,000. We computed all 100 eigenvalues with the real part in the interval [0, 50] (see Fig. 16). The relative residual tolerance was $10^{-6}$, the maximal subspace dimension 120, the slowest admissible convergence rate $\tau = 0.5$, $n_{locked} = 0$ and the automated restart parameters $\alpha =1$ and $N_{\alpha }=1$. Figure 17 shows the total CPU time and the time for solving of the projected quadratic eigenvalue problems. On average NRA method took 618.1 iterations and 11.1 restarts (12.1 LU factorizations) in 219.5 s, 67.4 s of which were spent on the solution of the projected linearized problems.

## Conclusions

We presented a local restart technique for iterative projection methods for solution of nonlinear Hermitian eigenvalue problems admitting a minmax characterization of their eigenvalues. We showed how the proposed technique can effectively eliminate a super-linear search subspace growth experienced when computing a large number of eigenvalues. Properly initialized, the method can be employed for computing eigenvalues in the interior of the spectrum. Iterative projection methods here considered work directly on the nonlinear eigenvalue problem without increasing its size and possibly destroying its structure by prior linearization. In this setting we do not have a transformation, like shift-invert for linear problems, mapping the eigenvalues close to a chosen shift to the exterior of the spectrum. In the absence of such transformation, spurious eigenvalues are intrinsic to interior eigenvalue computations and we proposed an effective strategy for dealing with such spurious values. We incorporated the proposed technique in the nonlinear iterative projection methods like the Nonlinear Arnoldi and Jacobi-Davidson methods. We illustrated various aspects of the local restart technique on numerical examples. The efficiency of the new restart framework was demonstrated on a range of nonlinear eigenvalue problems: three gyroscopic problems including a large gyroscopic eigenvalue problem modeling the dynamic behavior of a rotating tire, one exponential and one rational eigenvalue problem. Furthermore, we showed on two quadratic eigenvalue problems how the local restart technique can be extended to problems with complex eigenvalues with a dominant part (either real or imaginary). All the examples in this paper were solved using MATLAB toolbox QARPACK [2] containing an exemplary implementation of the locally restarted iterative methods ($\mathtt {qra}$: quadratic, $\mathtt {nra}$: general nonlinear solver). In the future we intend to extend the local restart technique to problems with more general distributions of the eigenvalues in the complex plane, close to a line or an a priori known curve.
